# Induction of Premature Cell Senescence Stimulated by High Doses of Antioxidants Is Mediated by Endoplasmic Reticulum Stress

**DOI:** 10.3390/ijms222111851

**Published:** 2021-10-31

**Authors:** Olga Lyublinskaya, Julia Kornienko, Julia Ivanova, Natalia Pugovkina, Larisa Alekseenko, Ekaterina Lyublinskaya, Irina Tyuryaeva, Irina Smirnova, Tatiana Grinchuk, Mariia Shorokhova, Anna Krasnenko, Nikolay Plotnikov, Nikolay Nikolsky

**Affiliations:** 1 Department of Intracellular Signaling and Transport, Institute of Cytology, Russian Academy of Sciences, Tikhoretskii pr. 4, 194064 St. Petersburg, Russia; kornienko.js@gmail.com (J.K.); ju.s.ivanova@yandex.ru (J.I.); natalia.pugovkina@gmail.com (N.P.); al.l.l@mail.ru (L.A.); katena.lyublinskaya@mail.ru (E.L.); inc_secretary@mail.ru (I.T.); sisfromspb@yahoo.com (I.S.); grintat@bk.ru (T.G.); shili-mariya@yandex.ru (M.S.); olga.lyublinskaya@incras.ru (N.N.); 2 Higher Engineering Physics School of the Institute of Physics, Nanotechnology and Telecommunications, Peter the Great St. Petersburg Polytechnic University, Polytechnicheskaya St. 29, 195251 St. Petersburg, Russia; 3 Genotek LLC, Nastavnicheskii per. 17/1, 105120 Moscow, Russia; krasnenko@genotek.ru (A.K.); Plotnikov@genotek.ru (N.P.)

**Keywords:** cell senescence, stress-induced senescence, antioxidants, mesenchymal stem cells, reductive stress, endoplasmic reticulum stress, replication stress, resveratrol, Tempol

## Abstract

In our previous study, we found that high doses of several substances with antioxidant capacities (Tempol, resveratrol, diphenyleneiodonium) can cause genotoxic stress and induce premature senescence in the human mesenchymal stem cells (MSCs). Here, using whole-transcriptome analysis, we revealed the signs of endoplasmic reticulum stress and unfolded protein response (UPR) in MSCs stressed with Tempol and resveratrol. In addition, we found the upregulation of genes, coding the UPR downstream target APC/C, and E3 ubiquitin ligase that regulate the stability of cell cycle proteins. We performed the molecular analysis, which further confirmed the untimely degradation of APC/C targets (cyclin A, geminin, and Emi1) in MSCs treated with antioxidants. Human fibroblasts responded to antioxidant applications similarly. We conclude that endoplasmic reticulum stress and impaired DNA synthesis regulation can be considered as potential triggers of cell damage and premature senescence stimulated by high-dose antioxidant treatments.

## 1. Introduction

Antioxidants (AOs) are usually understood as substances of natural or synthetic origin that can reduce the oxidative load in cells and tissues of an organism. In a stricter definition, an antioxidant (AO) is “any substance that delays, prevents, or removes oxidative damage to a target molecule” [1]. For quite a long time, pharmacologic AOs were believed to have an exclusively beneficial effect on the human body (reviewed in [2]). This belief was mainly based on the concept that oxidative processes accompanying aerobic respiration are the main reason for the accumulation of oxidative damage in cells, leading to the development of various pathologies and aging [3,4]. However, it has now become clear that oxidants generated by cells (commonly named reactive oxygen and nitrogen species, RONS) play an important regulatory role, participating in the vital metabolic and signaling pathways [5]. Cells carefully control RONS levels, preventing toxic thresholds from being exceeded. Taking this into account, the impact of the pharmacological AOs on cellular and organismic physiology is gradually reevaluated [6]. Accumulating experimental data indicate the negative effects of high-dose AO treatments on cells under normal physiological conditions [7,8,9,10]. Among these effects are inhibition of cell proliferation [11,12,13,14,15,16,17,18], DNA damage [19,20,21], apoptosis [20,21], and chromosomal abnormalities [19]. Sometimes, researchers obtain such data when analyzing the responses of cells to treatment with one single AO substance [11,13,15,16,22], and other times when testing a wide range of AOs [14,18,19,20]. Such observations allow classifying the discovered phenomena not as a side effect of one specific pharmacological agent but as a systemic disturbance arising from the AO overload. It is important to note that concentrations of AOs, which were found to be harmful to normal cells, are often used as the most protective in ex vivo experiments on oxidative stress-induced cell injury. It turns out that the administration of AOs at concentrations that rescue cells under oxidative stress conditions has a damaging effect on cells under normal physiological conditions.

In the past decade, in two independent studies [16,23], it has been found that resveratrol (a polyphenol of plant origin with antioxidant activity) produces opposite effects on normal cells when taken at different concentrations. Using human mesenchymal stem cell (MSC) cultures, it was shown that at low concentrations (less than 1 μM), resveratrol has a stimulating effect on cell proliferation. However, at higher concentrations (more than 5 μM) that are commonly used in ex vivo and in vitro studies and considered as pharmacologically potent [24], resveratrol has a cytostatic effect and can even cause premature senescence of cultured cells [16]. Moreover, in our latest study [25], we found that in addition to resveratrol, several other synthetic AOs with different chemical structures can stimulate senescence in cultured MSCs. We have shown that sub-cytotoxic doses of Tempol [26], resveratrol [27], and diphenyleneiodonium [28] cause disturbance of DNA replication in proliferating cells and lead to genotoxic effects and stress-induced premature senescence (SIPS). The results obtained are fundamental and have a significant practical import since the AOs are often thought of as geroprotective agents preventing aging both at the level of the whole organism and at the level of individual cells (reviewed in [29]).

In the present paper, we sought to move a step forward and investigate the potential molecular mechanisms of the negative impact of high-dose AO treatments on cells, assessing the transcription profiles of MSCs after Tempol and resveratrol treatments. Analyzing RNA sequencing data and validating these data using various molecular tests, we aimed to answer the following questions: (1) what the common features of transcriptional profiles after Tempol and resveratrol treatments are; (2) disturbance in which signaling/metabolic pathways accompanies the induction of the replication stress and senescence in AO-treated cells; and (3) what molecular mechanisms may underlie AO-induced stress.

## 2. Results

### 2.1. Induction of Stress after Tempol and Resveratrol Treatments

**Figure 1:**
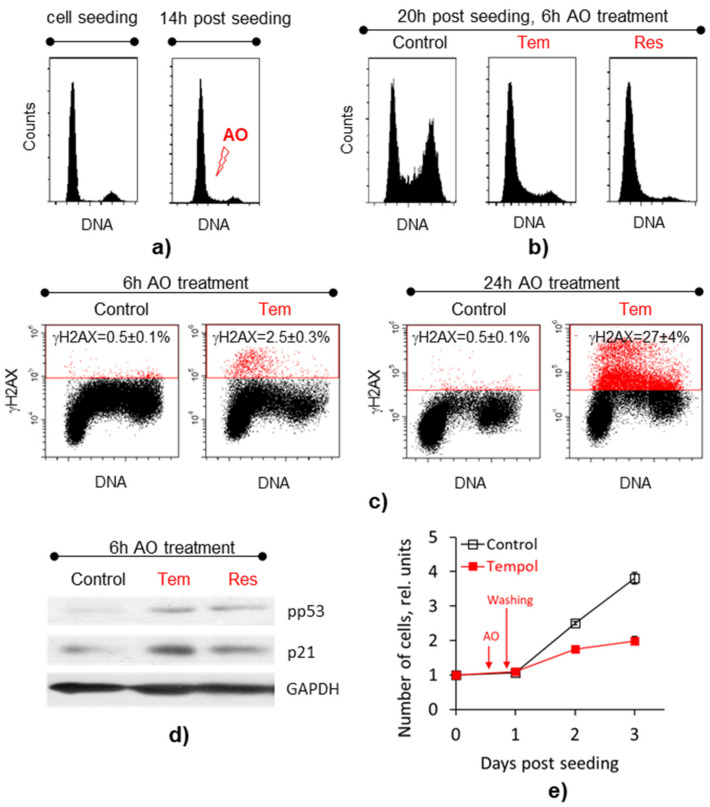
Cells treated with antioxidants in the early S phase slow down the DNA synthesis, accumulate DNA damage and lose proliferation capacity: (**a**) cell cycle distributions of synchronized MSCs at the moment of proliferation stimulation by reseeding (left panel) and at the moment of AO supplementation (right panel); (**b**) cell cycle phase distributions of the control MSCs and MSCs exposed to AOs for 6 h; (**c**) representative dot plots of the cell cycle distributions of MSCs stained with γH2AX antibodies after 6-h and 24-h incubations with Tempol (γH2AX-positive cells are marked with red color); (**d**) Western blot analysis of the pp53 and p21 protein expression after 6-h AO treatment; (**e**) cell growth curves for control MSCs, as well as MSCs incubated with Tempol for 6 h and then washed. Data (**c**,**e**) are shown as mean ± SD (*n* = 3). Abbreviations: antioxidants (AO), namely Tempol (Tem) and resveratrol (Res); human mesenchymal stem cells (MSCs), line#2804.

To investigate the transcription response of MSCs to the high-dose AO treatments, we stressed-cultured the MSCs using sub-cytotoxic concentrations of Tempol and resveratrol, following the procedure described in our previous study [25]. First, MSCs were synchronized in the G_0_/G_1_ phase of the cell cycle using high-density culturing (Figure 1a). Then, we stimulated cell proliferation by replating. Finally, at 14 h post-seeding, when the large fraction of the MSCs was in the late-G_1_/early-S phase of the cell cycle, 2 mM of Tempol or 40 µM of resveratrol were added to the cell medium. After 6-h incubation with AOs, we extracted the total RNA from the control and AO-treated cells to proceed with RNA sequencing. In the parallel experiments, we performed several molecular and functional tests to confirm stress induction in proliferating cells exposed to Tempol and resveratrol.

Our tests showed that in MSCs treated with AOs, the initiation and progression of the DNA synthesis phase slowed down compared to the control cultures. At the time of the RNA extraction, the cell cycle-synchronized AO-treated MSCs had not yet reached the G_2_/M phase of the cycle, while a significant part of the control MSCs had already reached it (Figure 1b). Immunofluorescence staining for the phosphorylated form of histone H2AX (γH2AX) showed that a minor cell fraction had double-stranded DNA breaks after 6-h AO treatments. However, after the 24-h incubation period, when cells finally reached the G_2_/M phase of the cycle, the fraction of γH2AX-positive MSCs increased significantly (Figure 1c and Figure S1 in the Supplementary Materials). Cells with γH2AX foci were detected exclusively in the S phase of the cycle.

In addition, after a 6-h treatment with AOs, we found an increase in the level of the phosphorylated form of the p53 protein and its underlying target p21 (Figure 1d). It is known that p53/p21 pathway mediates DNA damage response (DDR) by regulating the expression of genes implicated in the cell cycle arrest and initiation of premature cellular senescence program [30]. Finally, growth curves of the cells (Figure 1e) washed of AOs after a 6-h incubation indicated a decrease in their further proliferative activity, confirming the damaging effects of AO-induced stress. To prove the induction of premature senescence in MSCs after prolonged AO treatments, we investigated cell fate after AO withdrawal post-24-h exposure. Stressed MSCs retained viability but stopped dividing (Figure S2 in the Supplemental Materials).

In conclusion, the results were consistent with the previously obtained data [25] on the induction of replication disturbances, genotoxic effects, and cells’ premature senescence caused by sub-cytotoxic concentrations of Tempol and resveratrol.

### 2.2. Transcriptomic Analysis of Cells Treated with Tempol and Resveratrol Reveal Differentially Expressed Genes Common for Both AOs

**Figure 2:**
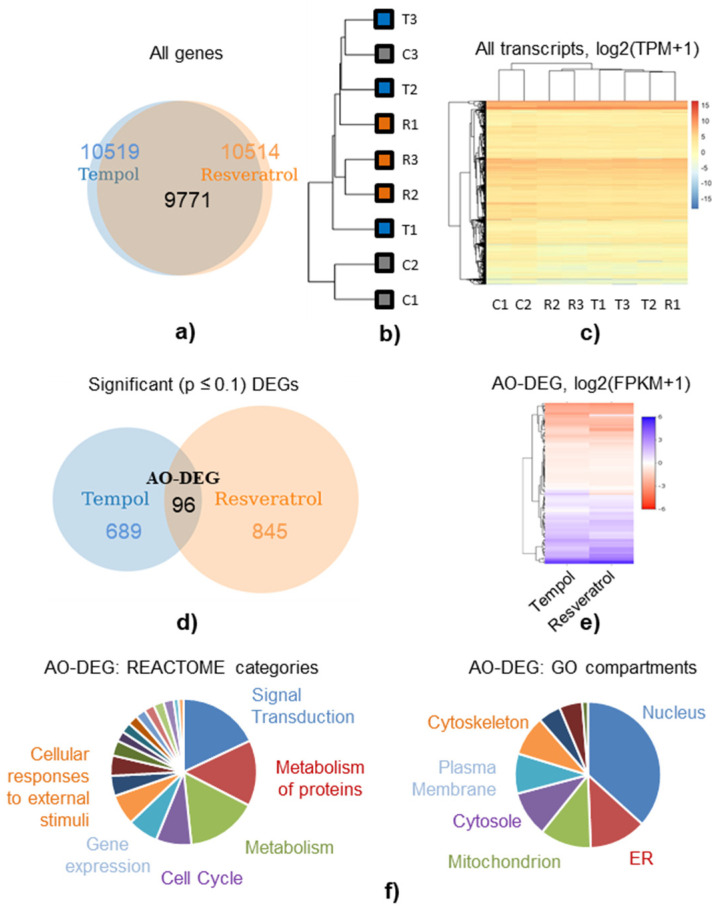
Transcriptomic analysis of the AO-stressed MSCs: differentially expressed genes; (**a**) genes identified with a nonzero expression in AO-stressed cells; (**b**,**c**) hierarchical clustering based on the log2(FPKM+1) of each gene (**b**) and on the log2(TPM+1) of each transcript (**c**); C, control RNA samples; T, RNA from Tempol-treated cells; R, RNA from resveratrol-treated cells; (**d**) significantly (*p*-value ≤ 0.1, Bonferroni correction) altered genes identified after AO treatments; intersection depicts AO-DEG set of genes (see also Table S1); (**e**) hierarchical clustering based on the log2(FPKM+1) of each gene from the AO-DEG list; (**f**) diagrams showing pathway core categories (REACTOME) and cellular compartments (GO) to which the genes from the AO-DEG list belong. Abbreviations: differentially expressed genes (DEGs); set of significant (*p*-value ≤ 0.1, Bonferroni correction) DEGs shared among both AO groups (AO-DEG); endoplasmic reticulum (ER); Fragments Per Kilobase Million (FPKM); Transcripts Per Kilobase Million (TPM); human mesenchymal stem cells (MSCs), line#2804.

Next, we performed RNA sequencing. We chose a 6-h time point to trace an early transcriptional response to the treatments with Tempol and resveratrol and focus on analyzing the causes of AO-induced stress rather than its consequences. After completing data analysis, we detected a nonzero expression of 10519 and 10514 genes in the cells treated with Tempol and resveratrol, respectively (Figure 2a). Based on the hierarchical clustering, one out of three control samples clustered with the AO-treated samples, while the other two control samples clustered in a separate group (Figure 2b,c). Despite one outlier sample in the control group, we did not exclude this sample from the analysis, and we identified differentially expressed genes (DEGs) based on all three biological replicates in order to have more statistically and biologically relevant results.

Among the genes identified with a nonzero expression in both Tempol- and resveratrol-treated groups (9771), only 96 genes appeared to be significantly (*p*-value ≤ 0.1, Bonferroni correction) differentially expressed in both groups, compared to the control samples (Figure 2d). Among those 96 genes, further referred to as the AO-DEG list (Table S1 in the Supplementary Materials), only six were oppositely regulated after Tempol and resveratrol applications. All the other 90 genes had the same expression patterns, indicating a strong similarity between transcriptional alterations common for both AOs (Figure 2e). The core categories (REACTOME) and cellular compartments (GO), to which these AO-DEG are referred, are presented in Figure 2f.

### 2.3. Transcriptomic Analysis Reveals Enriched Signaling and Metabolic Pathways Common for Both Tempol and Resveratrol Groups

**Figure 3:**
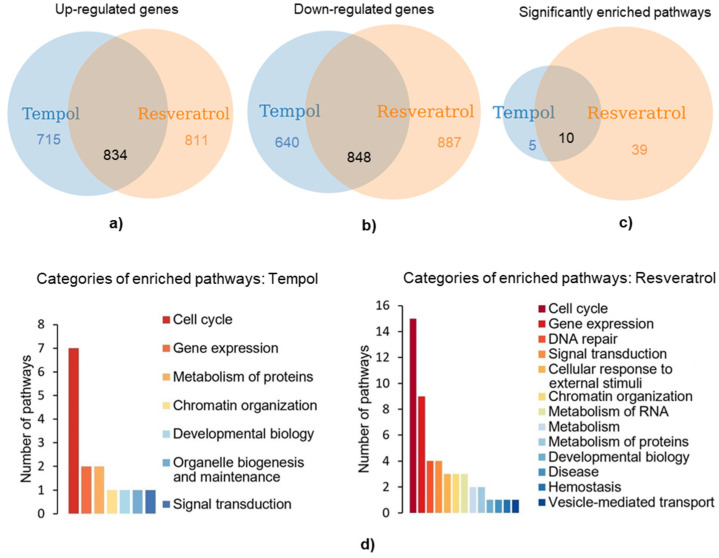
Transcriptomic analysis of AO-stressed MSCs: pathway enrichment analysis; (**a**,**b**) gene sets containing genes with an expression fold change increase or decrease greater than 1.5 used for pathway analysis; (**c**) Sets of pathways significantly (*p*-value ≤ 0.05, Benjamini and Hochberg analysis) enriched after AO treatments; (**d**) core categories to which the pathways enriched by each antioxidant belong. Abbreviations: human mesenchymal stem cells (MSCs), line#2804.

**Figure 4:**
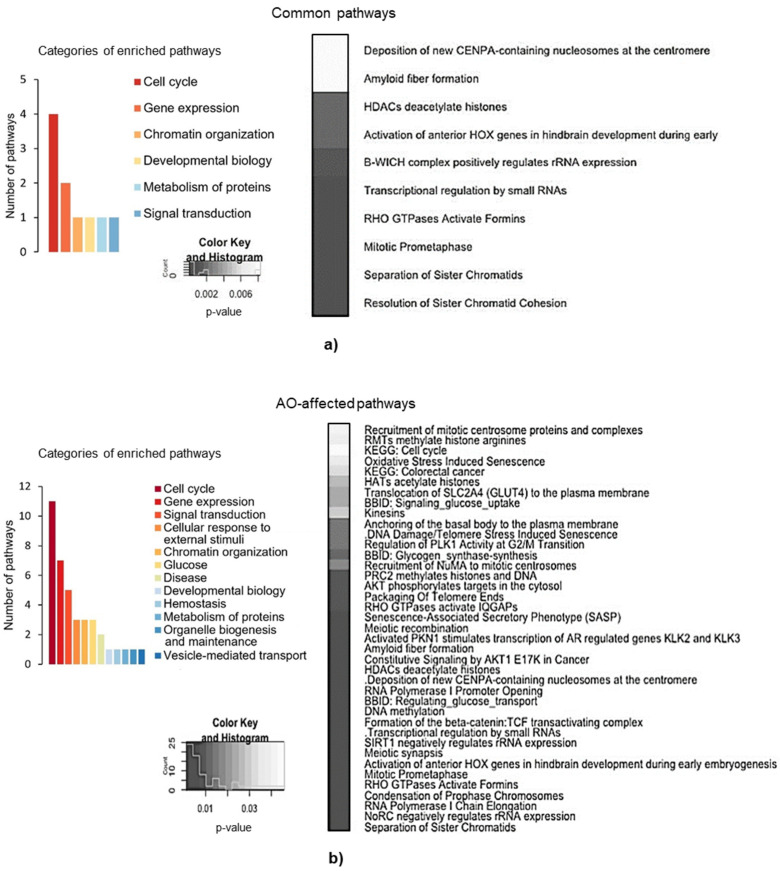
Common pathways enriched after Tempol and resveratrol treatments: (**a**) List of the core categories (left panel) to which the common pathways (right panel) significantly enriched by both antioxidants belong (*p*-value ≤ 0.05, Benjamini and Hochberg analysis); (**b**) list of the core categories (left panel) to which AO-affected pathways (right panel) belong. AO-affected pathways is a set of pathways significantly (*p*-value ≤ 0.05, Benjamini and Hochberg analysis) enriched by genes that are up- or down-regulated with a fold change greater than 1.5 by both AOs (such genes are shown as intersections of diagrams in Figure 3a,b).

Next, we pursued identifying pathways to which up- or down-regulated genes belong. To address this question, we utilized the sets of Tempol- and resveratrol-altered genes with an expression fold change increase or decrease of more than 1.5 (Figure 3a,b). We used the DAVID functional enrichment tool and looked only at pathways (BBID, BIOCARTA, EC_NUMBER, KEGG, REACTOME) and disease (OMIM). There were 15 and 49 pathways significantly (*p*-value ≤ 0.05, Benjamini and Hochberg analysis) enriched after the treatment with Tempol and resveratrol, respectively. Interestingly, among these pathways, ten were the same for cells treated with Tempol and resveratrol, which is two-thirds of all pathways enriched after the Tempol treatment (Figure 3c). Enriched pathway categories were similar between the AOs and are listed in Figure 3d (*Cell Cycle, Gene Expression, Signal Transduction, Chromatin Organization, Metabolism of Proteins*). As we were more interested in identifying common effects that the AOs have on the cellular transcriptome, we closely examined those ten pathways enriched after either of the treatments (Figure 4a).

Again, most of these pathways were related to the cell cycle regulation (more specifically mitosis) and others to transcription regulation, chromatin organization, and metabolism of proteins.

Next, we performed enrichment analysis in a different way. We used the set of commonly up- or down-regulated more than 1.5 times genes (Figure 3a,b only intersected gene lists) instead of previously used 1.5 up/down-regulated genes for each substance separately. The complete list of the statistically significant enriched pathways (further referred to as AO-affected pathways) is presented in Figure 4b. Most of these pathways were again functionally related to the cell cycle regulation, gene expression, and signal transduction. However, with this approach, some previously nonidentified pathways were captured, such as those belonging to the cellular response to the external stimuli (*DNA Damage/Telomere Stress Induced Senescence, Senescence-Associated Secretory Phenotype*), metabolism of glucose (BBID: *Signaling Glucose Uptake*, BBID: *Glycogen Synthase Synthesis*, BBID: *Regulating Glucose Transport*), disease (KEGG: *Colorectal Cancer*, *Constitutive Signaling by AKT1 E17K in Cancer*) (Figure 4b).

### 2.4. Transcriptomic Profiling Evidence of AO-Induced Slowdown of S Phase Progression, DNA Damage, and Premature Cell Senescence Induction

To verify the results of the cell transcriptome profiling, we focused on the interrelations between the enriched pathways and the phenotype of the AO-treated MSCs. The cell cycle phase distributions (Figure 1b) clearly indicated that at the time of the cell lysis for RNA extraction, a significant proportion of cells in the control cultures were in the G_2_/M phase, while cells treated with AOs in the early synthetic phase were still in the G_0_/G_1_ and S phases of the cycle. Accordingly, transcriptomic analysis shows that all signaling pathways related to mitosis were active in the control cells and suppressed in the cells incubated with AOs. Most of the signaling pathways (11 out of 39 in the list of AO-affected pathways, Figure 4b) enriched in AO-treated cells belong to the *Cell Cycle* category and are associated with the mitosis phase. Among the genes related to the *Cell Cycle,* many positive regulators of the DNA replication and mitosis (such as *TUBB, TUBB8, PLK1, CDC20, MLH3, LIN37*, set of replication-dependent histones, *CSNK2B, NUP85,* and others) are down-regulated. In contrast, many negative regulators of the DNA replication and mitosis (*WEE1, ANAPC4, ANAPC1, PDS5B, KIF18A, HERC2, DYRK1A, CDKN1C, CDCA5, ATR*, and others) are up-regulated (Figure S3 in the Supplementary Materials). The same trend is observed in the AO-DEG set. Thus, transcriptomic alterations confirmed observed earlier disturbance of S phase progression in AO-treated cells.

In addition to the impaired DNA synthesis, RNA sequencing confirms the induction of stress, DNA damage, and senescence in cells treated with AOs. Three signaling pathways from the list of AO-affected pathways belong to the subcategory of *Cellular Responses to External Stimuli/Cellular responses to Stress/Cellular Senescence* at once. Of these, one pathway (*DNA Damage/Telomere Stress Induced Senescence*) is associated with genotoxicity. In line with these results, the AO-DEG list also contains genes (*ANAPC4, CABIN1, HIST1H1E*) belonging to the *Cellular Senescence* subcategory. In addition, the AO-DEG list includes several genes that regulate the activity of the “genome guardian,” the transcription factor p53, whose activating phosphorylation was detected in the AO-treated cells by immunoblotting (Figure 1d). In the transcriptome of the AO-treated cells, negative regulators of p53 activity (*CABIN1, PLK1, CHD4*) are suppressed, while *ARHGAP11A* (a positive regulator) is up-regulated. The level of RNA of the *GADD45B* gene activated by the p53 factor is also significantly increased.

In conclusion, all the signs of replication disturbance and the cell damage that we revealed while performing the characterization of AO-treated MSCs (slowing of the S phase of the cell cycle, DNA damage, activation of p53, and predispositions for the senescence program activation) were also found in their transcription profiles, which verifies the results of the RNA sequencing performed.

### 2.5. Transcriptomic Profiling Reveals AO-Induced Effect on Redox-Dependent Signaling

In further analysis, we focused our search on the potential molecular triggers of genotoxic stress and premature senescence induced by high concentrations of AOs. Examination of the AO-DEG list showed that most of the genes from this list are involved in the pathways which belong to the *Signal Transduction* category. These genes are associated with Rho GTPase, Wnt and Akt signaling, signaling by receptor tyrosine kinases, and death receptor signaling (Table S1 in the Supplementary Materials). DEGs associated with Rho GTPase signaling (*ARHGAP11A, RTKN2, CTNNB1, PLK1, STARD13, NUP85, CDC20, GOPC*) are the most represented among them.

In the list of AO-affected pathways (Figure 4b), among the five pathways belonging to the *Signal Transduction* category, three paths are involved in the Rho GTPase-signaling, one in Wnt-signaling, and one in the Akt-signaling. The only enriched pathway in the *Disease* category also belongs to the *Akt Signaling* subcategory. Since Rho GTPase, Wnt, and *Akt-signaling* are proved to be redox-dependent cascades [31,32,33,34], it is not surprising that they mediate AO-induced stress and may be considered as its potential triggers.

### 2.6. Transcriptomic Profiling Reveals the Signs of AO-Induced ER Stress

**Figure 5:**
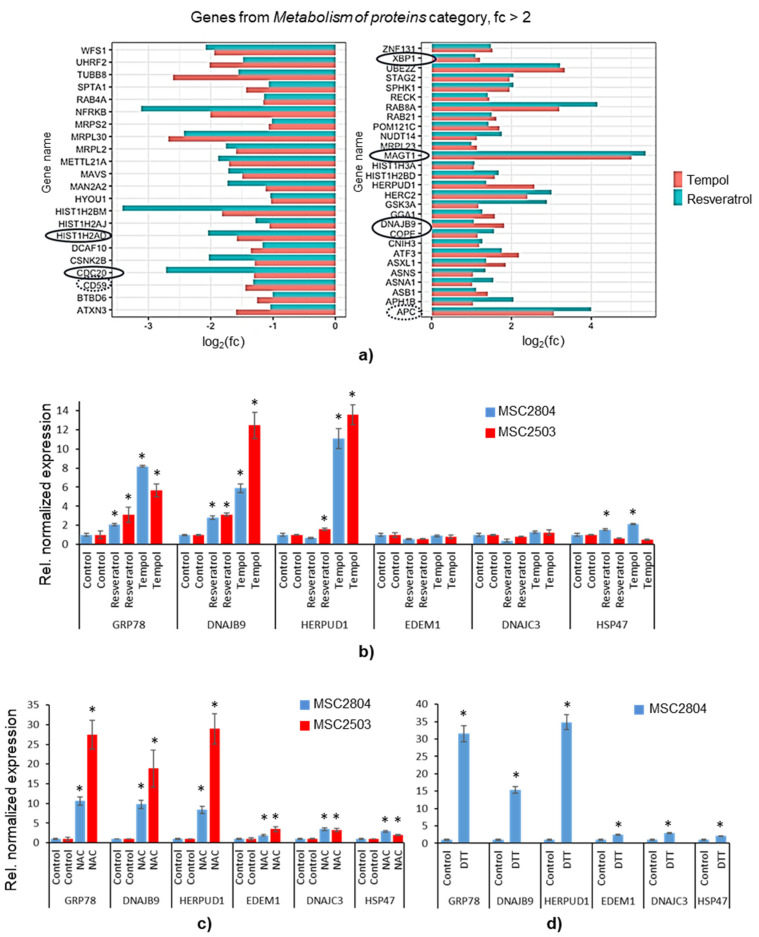
High-dose AO treatments induce ER stress and UPR: (**a**) sets of up- and down-regulated by both Tempol and resveratrol genes in the category *Metabolism of Proteins* with the fold change exceeding 2; significantly (*p*-value ≤ 0.1, Bonferroni correction) altered genes are marked; (**b**–**d**) Induction of ER stress and UPR marker genes upon MSC exposure to Tempol and resveratrol (**b**), NAC (**c**), DTT (**d**); the expression levels of the indicated genes were determined by real-time qPCR, normalizing against *Actin* expression, and calculated as fold difference from the control values; RNA was extracted from different MSC lines: MSC2804 (used throughout the study, blue color) and MSC2503 (red color). Data (**b**–**d**) are shown as mean ± SD. Two-tailed Students *t*-test was utilized for pairwise comparison. * *p* < 0.05 vs. corresponding control. Abbreviations: fold change of gene expression in Tempol- and resveratrol-treated cells (fc); endoplasmic reticulum (ER); unfolded protein response (UPR); dithiothreitol (DTT), N-acetyl-L-cysteine; (NAC); human mesenchymal stem cells (MSCs); lines#2804 and 2503.

In addition to the pathway analysis, we examined the cell compartments associated with genes from the AO-DEG set (Figure 2f). Most genes were involved in nuclear processes, which may reflect the nuclear localization of the proteins affected by AO-induced replication stress. However, the second most common localization of the genes with altered expression was in the endoplasmic reticulum (ER). The latter is consistent with a large number of genes in the AO-DEG list belonging to the pathways from *Metabolism of Proteins, Post-Translational Protein Modification,* and *Metabolism of Proteins/Unfolded Protein Response* subcategories. Overall, the *Metabolism of Proteins* category is the second most represented in the AO-DEG list. It is well known that disturbances in protein metabolism and folding cause ER stress, which, in turn, activates a set of signaling pathways termed the Unfolded Protein Response (UPR) [35]. We observed the AO-stimulated up-regulation of many genes involved in ER stress and UPR (Figure 5a).

In addition, analyzing the enriched pathways common to both AOs (Figure 4a,b), we found indirect evidence of ER stress, namely the suppression of the transcriptional and translational activity of the cells, which is a common consequence of the UPR. We noticed that the vast majority of enriched signaling pathways from *Chromatin Organization* and *Gene Expression* categories are associated with epigenetic mechanisms of gene activity regulation, such as acetylation-deacetylation and methylation of histones. All transcription activators from these categories (*ATF2, CLOCK, PBRM1, RUVBL1, POLR2D, GTF3C2, GTF2H2*) were down-regulated, while repressors (*SAP30L, DICER1*) were up-regulated (Figure S4 in the Supplementary Materials). Additionally, in the AO-DEG list (Table S1 in the Supplementary Materials), we found downregulation of two genes coding proteins from ribosomal 60S and 40S subunits, which may refer to the UPR-induced suppression of translation.

To verify the induction of ER stress and the UPR under the action of Tempol and resveratrol, we analyzed the expression of genes associated with ER stress in AO-treated cells using the qPCR method. We focused on the genes that, according to the published data [36], are the most reliable ER stressors and UPR markers (*GRP78, HERPUD1, EDEM1, DNAJC3, DNAJB9, HSP47*). According to the RNA-seq results, the expression of all these genes was increased after the treatments with the AOs. For qPCR, we used the RNA extracted for the sequencing experiment and RNA extracted in similar experiments with MSCs from a different donor (line MSC2503). We found (Figure 5b) that some of the ER stress-related genes were significantly (ten-fold) up-regulated in the cells of both lines (*GRP78, HERPUD1*, *DNAJB9*), while in the case of some markers, the response was insignificant (*HSP47*) or was entirely absent (*EDEM1, DNAJC3*).

In addition, we investigated the MSCs reaction to the treatment with N-acetylcysteine (NAC, 20 mM) to test whether other AOs can induce ER stress. NAC is a small molecule that possesses antioxidant activity due to its redox-active thiol groups and is a precursor of intracellular glutathione (GSH)—an essential element of antioxidant defense in all types of cells. In the MSCs treated with the NAC, the pattern of gene expression (Figure 5c) was similar to that induced by Tempol and resveratrol, but all the effects were significantly more pronounced, and all tested ER stress markers were up-regulated. It is known that NAC not only has a strong antioxidant effect but is also a potential inducer of reductive stress in cells [37]. Reductive stress, which is the counterpart to oxidative stress, is defined as an abnormal increase in the level of intracellular reducing equivalents in the form of NADH, NADPH, and GSH that results in various disturbances of cell metabolism, including ER stress and protein aggregation [38,39,40]. We hypothesized that excessive elimination of intracellular oxidants in MSCs caused by the action of Tempol and resveratrol could disrupt the redox balance and, similar to NAC, induce reductive stress. To assess the degree of overlap between the effects of AOs and reductive stress, we repeated our experiments using a potent reducing reagent dithiothreitol (DTT) instead of AOs. Treatment of cells with DTT (Figure 5d) resulted in the same gene expression pattern as incubations with AOs. This result indicated an inter-relation of the effects stimulated by high concentrations of AOs and reductive stress and pointed to reductive stress as one of the possible triggers of AO-induced stress. In conclusion, the RNA-seq results, verified by qPCR analysis, revealed the signs of ER-stress and UPR in AO-treated MSCs.

### 2.7. AO-Induced Stress Causes Destabilization of DNA Synthesis Regulators

In the following stage of our study, we focused on the relationship between ER stress and AO-induced disturbances of DNA replication. Since ER stress and UPR can themselves induce replication stress in cells [41], we aimed to identify genes from the AO-DEG list which can be both UPR targets and replication stress triggers. We drew attention to the *ANAPC4* gene (subunit of the Anaphase-promoting complex/cyclosome, APC/C) [42]. The APC/C, a ubiquitin E3-ligase, is the major effector protein complex responsible for the prevention of accumulation of S phase regulatory proteins in the early G_1_ phase of the cell cycle. In the late G_1_ phase, APC/C should be normally inactivated due to the activation of cyclin-dependent kinases (Cdks) and accumulation of APC/C inhibitor protein (early mitotic inhibitor-1 (Emi1)) [43]. Temporary APC/C inactivation, stimulated by Emi1 transitioning from acting as a substrate of APC/C to being its inhibitor [43], enables the accumulation of APC/C target proteins, such as cyclin A, geminin, and others, thus promoting proper S phase progression. It is known that both UPR [44] and a decrease in the physiological level of RONS under the action of various antioxidants [14] can induce untimely APC/C activity. Accordingly, having detected an increase in the amount of *ANAPC4* RNA in AO-treated cells, we hypothesized that the treatment of cells with AOs in the early S phase could lead to untimely activation of APC/C and degradation of DNA synthesis regulators, thereby inhibiting the progression of the synthetic phase of the cell cycle and causing replication stress. Along with *ANAPC4*, one more subunit of APC/C (*ANAPC1*) was up-regulated in Tempol- and resveratrol-treated cells, but with less than 0.9 significance (*p*-value > 0.1), adding additional support for our hypothesis.

**Figure 6:**
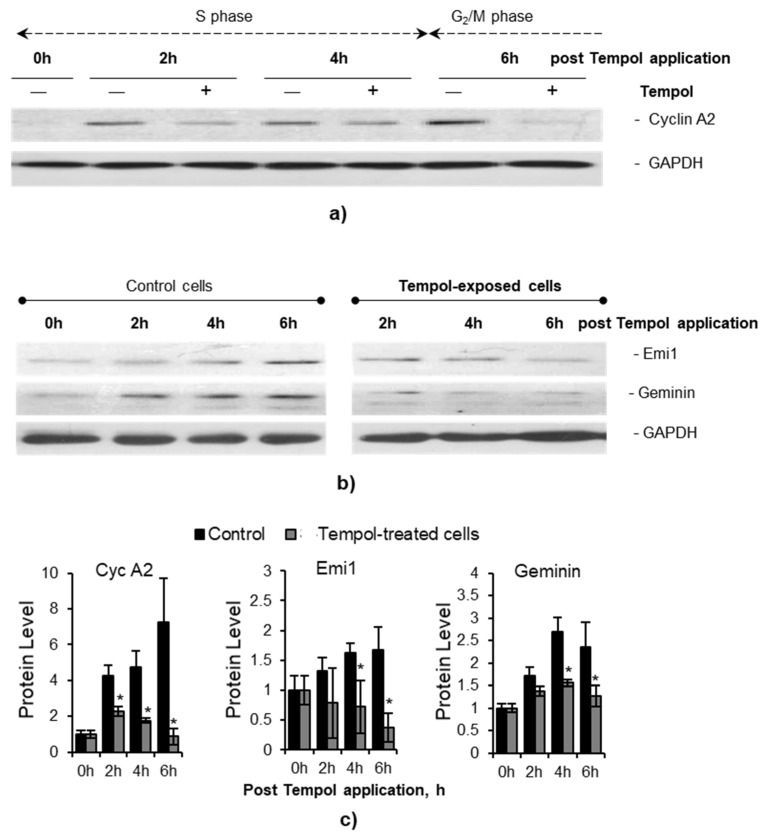
AO treatments performed in the S phase of the cell cycle cause decline in the level of cyclin A, geminin, and Emi1 proteins: (**a**–**c**) dynamics of the S phase regulatory protein level in control and Tempol-treated MSCs; representative immunoblot analysis for cyclin A2 (**a**), Emi1 (**b**) and geminin (**b**) proteins in the whole-cell lysates, as well as quantification of the protein bands (**c**) show the decline in the protein levels after Tempol application. Data (**c**) are normalized to the loading control (GAPDH) and calculated as fold difference from the initial value (average of three independent experiments ± SD); two-tailed Students *t*-test was utilized for pairwise comparison with the control values. * *p*-value ≤ 0.05. Abbreviations: human mesenchymal stem cells (MSCs), line#2804.

**Figure 7:**
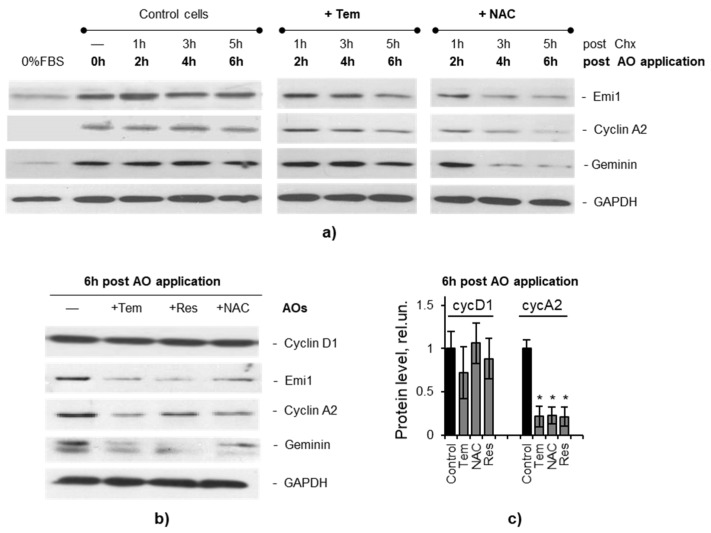
AO treatments cause instability of the key proteins regulating DNA synthesis: (**a**) dynamics of the regulatory protein’ (cyclin A2, Emi1, geminin) levels in the control and AO-exposed MSCs after protein translation inhibitor Chx addition to the cell medium (performed one hour after AO addition); representative immunoblot analysis of the whole-cell lysates shows the instability of the S phase regulatory proteins after AO application; (**b**,**c**) immunoblot analysis for cyclin A2, Emi1 and geminin proteins in the control and AO-treated cells lysed at 6 h post-AO addition; representative immunoblot (**b**) and quantification of the protein bands (**c**) reveal the low level of cyclin A2, Emi1 and geminin proteins in AO-treated cells; data are normalized to the loading control (GAPDH) and calculated as fold difference from the control values (average of four independent experiments ± SD); two-tailed Students *t*-test was utilized for pairwise comparison with the control values. * *p* < 0.05. Abbreviations: antioxidants (AOs), namely Tempol (Tem), resveratrol (Res), N-acetyl-L-cysteine (NAC); cycloheximide Chx; 0%FBS, serum-deprived MSCs (used as a negative control); human mesenchymal stem cells (MSCs), line#2804.

To test this hypothesis, we analyzed the dynamics of the levels of APC/C targets (cyclin A and geminin proteins) and the level of APC/C inhibitor (Emi1 protein) during the 6-h Tempol exposure of cell cycle-synchronized MSCs. The obtained data showed that, while the control cells were accumulating the said proteins during progression through the S phase of the cell cycle, their levels in Tempol-exposed cells were surprisingly decreasing (Figure 6). We then examined cyclin A, Emi1, and geminin stability in MSCs treated with Tempol. Both control and Tempol-exposed cells were treated with cycloheximide (applied 1 h post-Tempol addition to the cell medium) to inhibit protein synthesis. The investigated proteins were stable in the control cells, while in the Tempol-exposed MSCs, they were degraded after cycloheximide addition (Figure 7a). Consistent with these observations, the inhibition of the 26S proteasome with MG132 in the late S phase of the cell cycle resulted in the accumulation of geminin and partial recovery of the cyclin A level in Tempol-exposed cells (Figure S5 in the Supplementary Materials). We compared the effects of Tempol and resveratrol on the levels of cyclin A, geminin, and Emi1 proteins in MSCs after 6-h treatment. Our analysis showed that both AOs repressed accumulation of cyclin A, geminin, and Emi1 but did not affect the cyclin D, as the level of cyclin D is known to be independent of APC/C activity (Figure 7b,c). To determine whether other antioxidants can cause destabilization of the key regulators of DNA synthesis, we repeated our experiments with NAC and observed a similar effect to that of Tempol and resveratrol (Figure 7). We suggest that a possible cause of this effect may be a transcriptional activation of the APC/C subunits’ genes, as was shown by RNA-seq.

### 2.8. AOs Produce Similar Effects on Human MSCs and Fibroblasts

Finally, we investigated whether AO treatments could cause similar effects in normal human cells of a different origin. We compared the effects of Tempol, resveratrol, and NAC on the DNA synthesis progression and regulation in MSCs to those produced in human embryonic lung fibroblasts. We found that AOs, when applied to the cell-cycle synchronized fibroblasts in the early S phase, hampered S phase progression, led to the emergence of DNA strand breaks in cells replicating their DNA and disturbed cyclin A, geminin, and Emi1 accumulation (corresponding data are presented in the Figure S6 in the Supplementary Materials). Therefore, we observed a strong similarity in cell responses to the high-dose AO treatments in cultivated human MSCs and fibroblasts that point to the relevance of AO-induced stress for normal cell physiology.

## 3. Discussion

Our previous study [25] found that a 24-h treatment of proliferating, but not quiescent MSCs, with high but non-lethal doses of different AOs, leads to DNA damage, replication disturbances, and SIPS induction. In this study, we aimed at identifying whether the said AOs (Tempol and resveratrol), which have different origins and molecular targets, can similarly affect the MSC transcriptome when used at stressful concentrations. Surprisingly, we found much in common when comparing cell responses to both substances. Two-thirds of the signaling pathways enriched by Tempol were enriched in resveratrol-treated cells as well.

To verify the analysis performed, we first tracked the changes in the gene expression associated with the induction of replication stress and premature senescence. In the sets of significantly altered genes and pathways common for both AOs, we revealed transcriptional signs of stress-induced senescence, as well as DNA damage and slowdown in the synthetic phase progression, which are the most characteristic features of replication stress. Interestingly, the activation of genes associated with DNA damage and repair (Table S1 in the Supplementary Material) was observed as early as after 6-h incubation with AOs. At the same time, the γH2AX foci assay revealed DNA breaks at this time interval only in a small proportion of AO-treated cells. Since the fraction of cells with foci increased significantly at longer incubation times, we assume that the transcriptome changes found at the 6-h time point were caused by the DNA single-strand breaks, which can barely be detected using γH2AX assay. Generally, after the replication stress induction, single-strand break accumulation precedes the heavy DNA damage, but after prolonged stress conditions, the arrest and collapse of replication forks eventually lead to massive double-strand breaks [45]. Perhaps, in the cells treated with AOs, transcriptomic response anticipated these events. Similarly, activation of signaling pathways associated with cellular senescence preceded the development of phenotypic signs of SIPS, such as beta-galactosidase expression, cell enlargement, increased RONS levels, etc. According to our previously published data [25], these hallmarks begin to be detected several days after AO treatments. Therefore, the transcriptional response to replication stress predicts the further fate of AO-treated cells, apparently revealing the prerequisites for the induction of senescence, such as DNA damage. It is known that activation of SIPS programs is primarily associated with genotoxic effects [30].

Further, we focused on looking for possible triggers of AO-induced stress. We noticed first the enrichment of several redox-dependent pathways (Rho-GTPase, Wnt, AKT signaling) in AO-treated cells, with the most affected pathways from the subcategory of *Signaling by Rho GTPases*. It is known that proteins from the family of Rho GTPases contain redox-active cysteines in several conserved domains and hence are redox-regulated molecules [33]. Since AO-induced stress influenced signaling pathways involving different Rho GTPases, we classified GTPase-dependent signaling as Tempol- and resveratrol-sensitive. Perhaps future studies will reveal a more definite relationship between Rho GTPase signaling and AO-induced stress.

**Figure 8:**
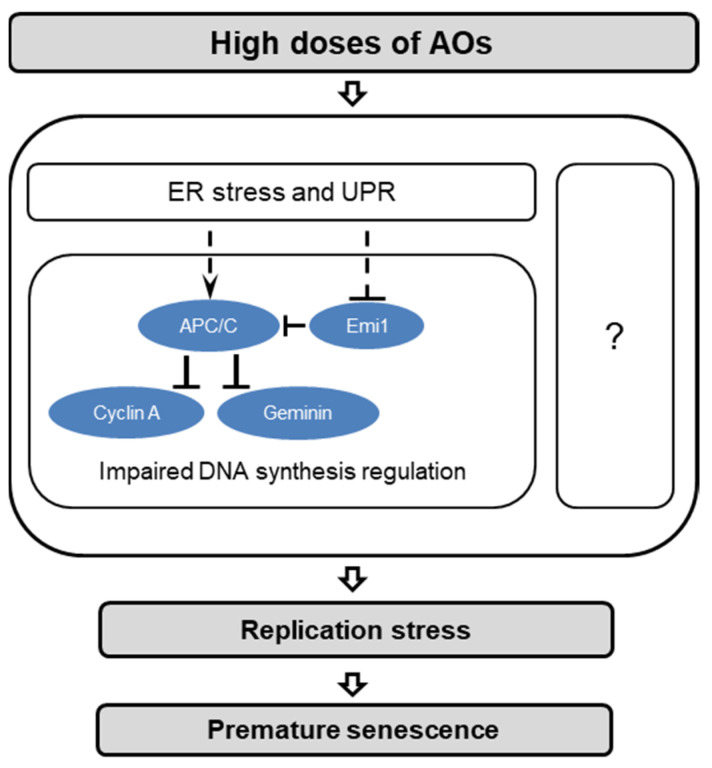
Effects caused by high-dose AO treatments. AO-induced stress can cause ER stress which is accompanied by disturbances in DNA synthesis regulation associated with the timeless activation of the APC/C ubiquitin ligase complex. The logic flow of the events (marked with dashed arrows) is hypothetical and based on the previously published data. A detailed description of the effects marked with grey panels can be found in [25]. The effect of AO-induced activation of APC/C marked with blue panels is discussed in [14].

The second most common category of signaling pathways, associated with the AO-DEG list, was the *Metabolism of Proteins* category. Analysis of this list revealed an upregulation of ER stress and UPR marker genes. The findings of the transcriptome analysis were verified using the qPCR method, which detected an increase in the expression of ER stress marker genes in cells treated with Tempol and resveratrol. Moreover, similar results were also obtained for cells treated with NAC, another potent AO. While low concentrations of AOs (e.g., NAC) have been previously found to reduce endoplasmic reticulum stress [46], high AO concentrations occur to produce opposite effects. It is known that NAC has a pronounced antioxidant capacity, exerting both a direct antioxidant effect on cells due to the presence of a free thiol group and indirect, being a precursor of cellular GSH [47]. Due to this property, NAC, especially at high (>5 mM) concentrations, can induce reductive stress in cells [37]. Reductive stress is a metabolic imbalance that occurs at the excess levels of intracellular reductants, including GSH [40]. Under these conditions, the redox equilibrium is shifted towards the reduction processes, i.e., in the direction opposite to oxidative stress. Since the maintenance of physiological redox homeostasis in the ER is critical for the functioning of enzymes responsible for proper protein folding, reductive stress can lead to protein misfolding and ER stress induction [39]. In our experiments, the expression pattern of the ER stress marker genes turned out to be very similar when MSCs were treated with both high concentrations of AOs (Tempol, resveratrol, and NAC) and DTT a classical inducer of reductive stress. Based on these observations and previously published data that confirm that resveratrol can induce ER stress [48], we hypothesize about the intersection of the effects induced by AOs and reductive stress. Of course, nothing is surprising in this, since the balance between the activity of the oxidative and reductive systems of the cell in favor of the latter can be shifted either by increasing the level of reductive equivalents, or vice versa, by lowering the level of oxidative ones. For example, it has been previously shown that chronic reductive stress can arise from the constitutive activation of the nuclear factor erythroid 2-like 2 (Nrf2), a master activator of antioxidant gene transcription [49]. Moreover, it is known that many AOs [50], including resveratrol [51], Tempol [52], and NAC [53], can themselves stimulate Nrf2 activity and/or expression. Therefore, we propose that it is acute reductive stress arising from the AO overload that serves as the source of AO-induced ER stress and the UPR (see scheme in Figure 8).

Finally, we focused on searching for downstream targets of UPR that can directly affect DNA replication. In the list of genes up-regulated by both AOs, we found several subunits of the APC/C protein complex. APC/C, a ubiquitin E3-ligase that controls cell cycle progression and can be activated upon induction of UPR [44]. APC/C is responsible for the degradation of key proteins that regulate DNA replication (cyclin A, geminin, etc.) and is ordinarily inactive during the synthetic phase of the cycle, partly due to the accumulation of the Emi1 protein that inhibits APC/C [42]. We hypothesized that the upregulation of genes encoding APC/C subunits could be a link between the UPR and the induction of replication stress in cells treated with Tempol and resveratrol (Figure 8). The performed molecular analysis confirmed the instability of APC/C targets (cyclin A, geminin) and its inhibitor Emi1 in AO-treated cells, lending further support to our hypothesis. Moreover, we found that the treatment of cells with NAC also destabilized these regulators of DNA synthesis. Additionally, high concentrations of AOs were proved to cause the disturbances in cyclin A, geminin, and Emi1 accumulation not only in MSCs but also in human embryonic fibroblasts, pointing to the relevance of AO-induced stress for normal cell physiology.

It should be noted that the effect of AO-induced destabilization of cyclin A and Emi1 caused by untimely activation of APC/C was first discovered by Havens et al. [14]. Havens and colleagues showed that this effect is responsible for blocking AO-treated cells at the G_1_-S phase boundary, inhibiting the initiation of the DNA synthesis phase. In the present study, we observed an effect of destabilization of the APC/C targets in the cells treated with AOs at the early S phase. In this case, APC/C activation was accompanied by inhibition of the DNA synthesis and induction of replication stress. AO-induced replication stress, in turn, leads to DNA damage and activation of the SIPS programs (see scheme in Figure 8). Taken together, these observations underline the central role of APC/C in the regulation of cell proliferation and allow considering this complex not only as a regulator of sequential changes in the cell cycle phases but also as a universal tool for blocking the cycle outside of its control points.

The data obtained in this study do not allow us to reliably determine the causal relationship between ER stress, destabilization of DNA synthesis regulators, and replication stress in AO-treated cells. We can only state that these effects mediate AO-induced stress and SIPS and postulate a relationship between these processes (see scheme in Figure 8), based on the previously published data [14,42,44]. Importantly, even if the predicted logic flow of the AO-stimulated effects is not entirely correct, the results of our study allow outlining the range of possible causes of AO-induced stress. Further studies, including loss- or gain-of-function experiments, are needed to distinguish between causative and consequential effects of high-dose AO treatments. In future studies, we also hope to reveal a more definitive relationship between the AO-stimulated and reductive stress and, finally, answer whether acute reductive stress underlies AO-induced replication stress and cellular senescence.

One more critical issue that nevertheless needs clarification concerns the AO concentrations used in our study. First, it is important to note that these concentrations cannot be found in antioxidant-rich foods, so it is pointless to link our findings to dietary recommendations. In the case of Tempol, the concentrations we used were several times higher than those typically used in cell studies (including those recently applied to target SARS-CoV-2 infection [54]). Nevertheless, at the same time, the chosen concentrations of resveratrol and NAC exactly match the range that is widely used in ex vivo and in vitro experiments. These concentrations do not cause cytotoxic effects and are commonly considered most effective [24]. In clinical trials of resveratrol in humans, different doses are used from tens of milligrams to several grams at a time. On the one hand, even at the high dose of 5 g, which was used in phase I trials on healthy volunteers [55], the detected concentration of resveratrol in the blood (958.6 μg/L, which corresponds to 4.2 μM) is an order of magnitude lower than that used in the present research. However, there is evidence [56] that the repeated administration of the drug causes the accumulation of resveratrol in the tissues of patients at concentrations of up to 674 nmoL/g. When replacing grams for milliliters, the latter is much higher than the concentration of 40 μM, which was used in the present study. Thus, our findings prove that the use of AOs in clinical trials or as a biologically active additive should be better tested and strictly controlled.

## 4. Conclusions

We have shown that replication stress induced in the MSC cultures by high doses of antioxidants (Tempol and resveratrol) is accompanied by the upregulation of genes associated with stress, DNA damage, and cellular senescence and affects pathways associated with redox-dependent signaling, ER stress, and UPR. The analysis of qPCR verified the upregulation of ER stress marker genes in MSCs treated with Tempol and resveratrol. A similar pattern of marker gene expression was found after treating cells with the reductive stress inducers (NAC, DTT), indicating a similarity of the effects caused by AO-induced and reductive stresses. In the transcriptome of cells treated with Tempol and resveratrol, upregulation of genes coding the downstream target of UPR and ubiquitin E3-ligase APC/C was observed. The molecular analysis of cell-cycle synchronized MSCs treated in the early phase of DNA synthesis with AOs (Tempol, resveratrol, NAC) revealed the instability of APC/C targets, vital regulators of DNA replication (cyclin A, geminin), as well as APC/C inhibitor (Emi1). We conclude that ER stress and impaired DNA synthesis regulation mediate AO-induced stress and can be considered potential triggers of cell damage and premature senescence stimulated by high-dose AO treatments.

## 5. Materials and Methods

### 5.1. Cell Cultures

Two lines (MSC2804 and MSC2503) of human mesenchymal stem cells were derived from a desquamated endometrium of menstrual blood from healthy donors following the procedure described previously [54]. MSCs are clonogenic, express CD13, CD29, CD44, CD73, CD90, and CD105 surface markers, co-express CD146 and CD140 markers, are negative for the hematopoietic markers CD34 and CD45 and can be differentiated into osteoblasts and adipocytes [57]. MSCs were cultivated in DMEM/F12 growth medium containing 10% fetal bovine serum (FBS, HyClone, Logan, UT, USA), 1% L-glutamine (Gibco, Thermo Fisher Scientific, Amarillo, TX, USA), and 1% penicillin-streptomycin (Gibco, Thermo Fisher Scientific, USA). Cells were maintained in 75 cm^2^ culture flasks at 37 °C in a humidified chamber with 5% CO_2_ and subcultured twice per week. The MSCs of the 8th–9th passage have been used for performing experiments. MSC2804 line was used in all experiments described in the study, MSC2503 for qPCR analysis only.

Human lung fibroblasts were obtained from the Cell collection of the Research Institute of Influenza (St. Petersburg, Russia). Cells were cultivated in DMEM/F12 growth medium containing 10% fetal bovine serum (FBS, HyClone, Logan, UT, USA), 1% L-glutamine (Gibco, Amarillo, TX, USA), and 1% penicillin-streptomycin (Gibco, Thermo Fisher Scientific, USA). Cells (from the 20th to 30th passages) were maintained in 75 cm^2^ culture flasks at 37 °C in a humidified chamber with 5% CO_2_ and subcultured twice per week.

### 5.2. Cell Treatments

Within the study, we used three different synthetic substances with AO capacity (resveratrol, Tempol, and NAC). Tempol (4-Hydroxy-TEMPO, Santa Cruz Biotechnologies, USA) is a low molecular weight redox-cycling nitroxide, a superoxide dismutase mimetic, and free radical scavenger [26]. Resveratrol (3,4′,5-trihydroxystilbene, Sigma, Merck, Kenilworth, NJ, USA) is a natural polyphenolic phytoalexin, free radical scavenger, and antioxidant enzymes activity promoter [58]. NAC (N-acetyl-L-cysteine, Sigma, Merck, USA) is an aminothiol precursor of intracellular cysteine and reduced glutathione (GSH) and free radical scavenger [47]. To prepare the stock solutions, Tempol was dissolved in distilled water at a concentration of 1 M, and resveratrol was dissolved in DMSO at a concentration of 100 mM. Working solutions (100 mM and 1 mM for Tempol and resveratrol, respectively) were prepared before applications by diluting stock solutions in a fresh culture medium. NAC working solution (1 M in water) was adjusted for pH 7.4 and used freshly prepared.

To investigate AO-induced replication stress, we used cell cultures synchronized in the cell cycle. Synchronization was achieved either by high-density culturing or by serum deprivation, as indicated. After collecting cells in the G_0_/G_1_ phase of the cell cycle, cells were stimulated for proliferation either by reseeding or changing the growth medium to FBS-supplemented one. Both methods of synchronization yielded similar results. Serum deprivation has been used in Western blotting experiments to manage their time schedule better. Resveratrol at 40 μM, NAC at 20 mM, and Tempol at 2 mM concentrations were applied to cells 14 h after stimulation of cell proliferation. Dithiothreitol (DTT, Sigma, Merck, USA) at a final concentration of 1 mM was applied in the same way. All substances were constantly present in the cell medium until the analysis.

For protein stability analysis, cells were treated with either protein translation inhibitor cycloheximide (Chx, final concentration of 700 µM) or 26S proteasome inhibitor MG132 (final concentration of 1 nM). Chx (Sigma, Merck, USA) was added to the growth medium 1 h after AO addition. MG132 (Sigma, Merck, USA) was added 4 h after AO addition.

### 5.3. RNA Sequencing

In three independent experiments, total RNA was extracted from the control and treated for 6 h with Tempol or resveratrol cells. Extraction was performed using ExtractRNA kit (Evrogen, Moscow, Russia) according to the manufacturer’s instructions. For cDNA synthesis, the Mint-2 kit (Evrogen, Russia) was applied. Libraries have been prepared with the use of New England Biolabs NEBNext^®^ Ultra™ DNA Library Prep Kit for Illumina^®^ according to the manufacture protocols. After the denaturation and dilution, samples were sequenced in the rapid run mode on the Illumina HiSeq 2500 platform. Datasets were generated in the FASTQ format.

### 5.4. Transcriptome Analysis

Sequencing reads were processed and aligned to the human reference genome (hg19/GRCh37) with the HISAT2 program (v.2.1.0), gene coverages were calculated with Stringtie, and then analysis was performed with the Ballgown software package the pipeline explained in [https://www.nature.com/articles/nprot.2016.095#t3, accessed on 3 October 2021].

### 5.5. Cell Cycle Analysis

Cells were harvested with 0.05% trypsin-EDTA solution, suspended in the fresh medium, permeabilized with 0.1% Triton X-100 (Sigma, Merck, USA), and stained for 5 min with 2 µg/mL of 4′,6-diamidino-2-phenylindole (DAPI, Sigma, Merck, USA). Cell-cycle phase distribution was measured with CytoFLEX flow cytometer (Beckman Coulter, Brea, CA, USA, 405 nm laser) and analyzed using CytExpert 2.0 software.

### 5.6. Cell Viability Analysis

Cells, harvested with 0.05% trypsin-EDTA solution and suspended in the fresh medium, were stained with 50 μg/mL propidium iodide (Sigma-Aldrich, Merck, USA) and analyzed with CytoFLEX flow cytometer (Beckman Coulter, USA, 488 nm laser).

### 5.7. DNA Damage Assay

Cells, harvested with 0.05% trypsin-EDTA solution and suspended in the fresh medium, were washed twice with PBS, fixed, and permeabilized using Nuclear Factor Fix and Perm Buffer Set (BioLegend, San Diego, CA, USA). For specific detection of γH2AX foci accumulation, cells were incubated with anti-γH2AX antibodies (1:200, Abcam, Tokyo, Japan) for 1 h at room temperature in the dark. After washing with PBS, cells were incubated with 1:500 solution of goat-anti-mouse (GAM) Alexa Fluor^®^ 488 secondary antibodies and 1 µg/mL of DAPI for 30 min at room temperature in the dark. The γH2AX foci accumulation, as well as its distribution among the cell cycle phases, was then analyzed with CytoFLEX flow cytometer (Beckman Coulter, USA, 405/488 nm lasers). MSCs treated with hydrogen peroxide (200 µM for 1 h) were used as a positive control (see Supplemental Figure S1).

### 5.8. Western Blotting

Cells were lysed as was described previously (6). Protein samples were separated by either 4% or 12%-SDS-polyacrylamide gel electrophoresis (SDS-PAGE) with subsequent transfer onto a 0.45 µm nitrocellulose membrane (BioRad, Tokyo, Japan). Blots were blocked with 5% nonfat dry milk in Tris-buffered saline supplemented with 0.05% Tween 20 for 1 h at room temperature and then incubated overnight at 4 °C with following primary antibodies: anti-phospho-pRb (Ser807/811, 1:1000), anti-cyclin A2 (1:2000), anti-glyceraldehyde 3-phosphate dehydrogenase (GAPDH, 1:1000), anti-geminin (1:1000), anti-phospho-p53 (Ser15, 1:1000), anti-p21Waf1/Cip1 (1:1000), anti-phospho-ATM (Ser1981, 1:1000), anti-cyclin D1 (1:1000, all from Cell Signaling Technology, Danvers, MA, USA), and anti-Emi1 (1:1000, Abcam, USA). Blots were washed, incubated with peroxidase-conjugated goat anti-mouse IG (GAM-HRP, 1:10,000) and goat anti-rabbit IG (GAR-HRP, 1:10,000, both from Cell Signaling Technology, USA) for 1 h at room temperature and developed with ECL (Thermo Scientific, USA). Hyperfilm (CEA) was from Amersham. For densitometric analysis of protein bands ImageJ software (US National Institutes of Health) was used.

### 5.9. qPCR Assays

To analyze gene expression, total RNA was isolated with RNeasy Micro Kit (Qiagen, Hilden, Germany) according to the manufacturer’s instructions. RNA was quantified in the NanoDrop ND-1000 Spectrophotometer (Thermo Fisher Scientific, Waltham, MA, USA). We obtained cDNA by reverse transcription of RNA using the RevertAid H Minus First Strand cDNA Synthesis Kit (Fermentas, Waltham, MA, USA) according to the manufacturer’s instructions. The cDNA was amplified with specific primers, using EvaGreen^®^ dye (Biotium) and Dream-Taq™ PCR Master Mix (2X) (Thermo Fisher Scientific, Waltham, MA, USA) in the Bio-Rad CFX-96 real-time system (Bio-Rad, Tokyo, Japan), according to the kit’s enclosed protocol. Expression of target genes was normalized to *Actin*. Primers are presented in Table S2. Reaction conditions were: 93 °C 20 s, 60 °C 20 s, 72 °C 30 s.

### 5.10. Statistical Analysis

All data are presented as the mean values of at least three independent experiments with standard deviations. Statistical significance was calculated using either the ANOVA-Tukey test for multiple comparisons or the Student’s *t*-test in case of pair comparisons. *p*-value ≤ 0.05 (or ≤0.1, if indicated) were considered significant. To set the AO-DEG and AO-affected pathways lists, Bonferroni correction or Benjamini and Hochberg (BH) analysis have been applied, respectively.

## Data Availability

The data presented in this study are available on request from the corresponding author.

## Supplementary Materials

The following are available online at www.mdpi.com/article/10.3390/ijms222111851/s1, Figure S1: MSCs treated with resveratrol in the early S phase of the cycle accumulate DNA breaks, Figure S2: MSCs treated with AOs in the early S phase of the cycle preserve viability but lose proliferative capacity, Figure S3: AO treatments result in the downregulation of DNA replication, and mitosis regulators, Figure S4: AO treatments result in the downregulation of transcription activators and upregulation of transcription repressors, Figure S5: Tempol treatments cause instability of the key regulatory proteins of the S phase, Figure S6: Fibroblasts treated with AOs in the S phase of the cell cycle respond to AO treatments in the similar to MSC cultures way, Table S1: Set of significant (*p*-value ≤ 0.1, Bonferroni correction) differentially expressed genes common for Tempol and resveratrol groups (referred to as AO-DEG list), Table S2: Primers used for qPCR analysis.

## References

[1] Halliwell B., Gutteridge J.M.C. (2015). Free Radicals in Biology and Medicine.

[2] Neha K., Haider M.R., Pathak A., Yar M.S. (2019). Medicinal prospects of antioxidants: A review. Eur. J. Med. Chem..

[3] Harman D. (1969). Prolongation of life: Role of free radical reactions in aging. J. Am. Geriatr. Soc..

[4] Harman D. (1956). Aging: A theory based on free radical and radiation chemistry. J. Gerontol..

[5] Sies H., Jones D.P. (2020). Reactive oxygen species (ROS) as pleiotropic physiological signalling agents. Nat. Rev. Mol. Cell Biol..

[6] Schmidt H.H.H.W., Stocker R., Vollbracht C., Paulsen G., Riley D., Daiber A., Cuadrado A. (2015). Antioxidants in Translational Medicine. Antioxid. Redox Signal..

[7] Dundar Y., Aslan R. (2000). Antioxidative stress. East. J. Med..

[8] Villanueva C., Kross R.D. (2012). Antioxidant-induced stress. Int. J. Mol. Sci..

[9] Gostner J.M., Becker K., Ueberall F., Fuchs D. (2015). The good and bad of antioxidant foods: An immunological perspective. Food Chem. Toxicol..

[10] Poljsak B., Milisav I. (2012). The neglected significance of “antioxidative stress”. Oxid. Med. Cell. Longev..

[11] Kim K.Y., Rhim T.Y., Choi I., Kim S.S. (2001). N-Acetylcysteine Induces Cell Cycle Arrest in Hepatic Stellate Cells through Its Reducing Activity. J. Biol. Chem..

[12] Gamalei I.A., Polozov I.S., Kirpichnikova K.M., Aksenov N.D., Tararova N.D., Pospelova T.V. (2001). Distribution of rat embryonal fibroblasts through cell cycle phases in the presence of inhibitors of active oxygen species formation and N-acetylcysteine. Tsitologiia.

[13] Scaife R.M. (2004). G2 cell cycle arrest, down-regulation of cyclin B, and induction of mitotic catastrophe by the flavoprotein inhibitor diphenyleneiodonium. Mol. Cancer Ther..

[14] Havens C.G., Ho A., Yoshioka N., Dowdy S.F. (2006). Regulation of Late G1/S Phase Transition and APCCdh1 by Reactive Oxygen Species. Mol. Cell. Biol..

[15] Menon S.G., Sarsour E.H., Kalen A.L., Venkataraman S., Hitchler M.J., Domann F.E., Oberley L.W., Goswami P.C. (2007). Superoxide signaling mediates N-acetyl-L-cysteine-induced G1 arrest: Regulatory role of cyclin D1 and manganese superoxide dismutase. Cancer Res..

[16] Peltz L., Gomez J., Marquez M., Alencastro F., Atashpanjeh N., Quang T., Bach T., Zhao Y. (2012). Resveratrol exerts dosage and duration dependent effect on human mesenchymal stem cell development. PLoS ONE.

[17] Paul M.K., Bisht B., Darmawan D.O., Chiou R., Ha V.L., Wallace W.D., Chon A.T., Hegab A.E., Grogan T., Elashoff D.A. (2014). Dynamic changes in intracellular ROS levels regulate airway basal stem cell homeostasis through Nrf2-dependent notch signaling. Cell Stem Cell.

[18] Lyublinskaya O.G., Borisov Y.G., Pugovkina N.A., Smirnova I.S., Obidina J.V., Ivanova J.S., Zenin V.V., Shatrova A.N., Borodkina A.V., Aksenov N.D. (2015). Reactive oxygen species are required for human mesenchymal stem cells to initiate proliferation after the quiescence exit. Oxid. Med. Cell. Longev..

[19] Li T.S., Marbán E. (2010). Physiological levels of reactive oxygen species are required to maintain genomic stability in stem cells. Stem Cells.

[20] Fox J.T., Sakamuru S., Huang R., Teneva N., Simmons S.O., Xia M., Tice R.R., Austin C.P., Myung K. (2012). High-throughput genotoxicity assay identifies antioxidants as inducers of DNA damage response and cell death. Proc. Natl. Acad. Sci. USA.

[21] Lu L.Y., Ou N., Lu Q. (2013). Bin Antioxidant Induces DNA damage, cell death and mutagenicity in human lung and skin normal cells. Sci. Rep..

[22] Longpre J.M., Loo G. (2008). Paradoxical effect of diphenyleneiodonium in inducing DNA damage and apoptosis. Free Radic. Res..

[23] Demidenko Z.N., Blagosklonny M.V. (2009). At concentrations that inhibit mTOR, resveratrol suppresses cellular senescence. Cell Cycle.

[24] Vang O. (2013). What is new for resveratrol? Is a new set of recommendations necessary?. Ann. N. Y. Acad. Sci..

[25] Kornienko J.S., Smirnova I.S., Pugovkina N.A., Ivanova J.S., Shilina M.A., Grinchuk T.M., Shatrova A.N., Aksenov N.D., Zenin V.V., Nikolsky N.N. (2019). High doses of synthetic antioxidants induce premature senescence in cultivated mesenchymal stem cells. Sci. Rep..

[26] Wilcox C.S. (2010). Effects of tempol and redox-cycling nitroxides in models of oxidative stress. Pharmacol. Ther..

[27] Bhat K.P.L., Kosmeder J.W., Pezzuto J.M. (2001). Biological effects of resveratrol. Antioxid. Redox Signal..

[28] Li Y., Trush M.A. (1998). Diphenyleneiodonium, an NAD(P)H oxidase inhibitor, also potently inhibits mitochondrial reactive oxygen species production. Biochem. Biophys. Res. Commun..

[29] Fusco D., Colloca G., Lo Monaco M.R., Cesari M. (2007). Effects of antioxidant supplementation on the aging process. Clin. Interv. Aging.

[30] Campisi J., D’Adda Di Fagagna F. (2007). Cellular senescence: When bad things happen to good cells. Nat. Rev. Mol. Cell Biol..

[31] Zhang J., Wang X., Vikash V., Ye Q., Wu D., Liu Y., Dong W. (2016). ROS and ROS-Mediated Cellular Signaling. Oxid. Med. Cell. Longev..

[32] Funato Y., Miki H. (2010). Redox regulation of Wnt signalling via nucleoredoxin. Free Radic. Res..

[33] Mitchell L., Hobbs G.A., Aghajanian A., Campbell S.L. (2013). Redox regulation of ras and rho GTPases: Mechanism and function. Antioxid. Redox Signal..

[34] Ferro E., Goitre L., Retta S.F., Trabalzini L. (2012). The Interplay between ROS and Ras GTPases: Physiological and Pathological Implications. J. Signal. Transduct..

[35] Hetz C., Papa F.R. (2018). The Unfolded Protein Response and Cell Fate Control. Mol. Cell.

[36] Gupta S., Samali A., Fitzgerald U., Deegan S. (2010). Methods for monitoring endoplasmic reticulum stress and the unfolded protein response. Int. J. Cell Biol..

[37] Kolossov V.L., Beaudoin J.N., Ponnuraj N., Diliberto S.J., Hanafin W.P., Kenis P.J.A., Gaskins H.R. (2015). Thiol-based antioxidants elicit mitochondrial oxidation via respiratory complex III. Am. J. Physiol Cell Physiol.

[38] Quiles J.M., Narasimhan M., Mosbruger T., Shanmugam G., Crossman D., Rajasekaran N.S. (2017). Identification of transcriptome signature for myocardial reductive stress. Redox Biol..

[39] Narasimhan K.K., Devarajan A., Karan G., Sundaram S., Wang Q., van Groen T., del Monte F., Rajasekaran N.S. (2020). Reductive stress promotes protein aggregation and impairs neurogenesis. Redox Biol..

[40] Rajasekaran N.-S. (2020). Reductive Stress: Neglected Science. Antioxid. Redox Signal..

[41] Cabrera E., Hernández-Pérez S., Koundrioukoff S., Debatisse M., Kim D., Smolka M.B., Freire R., Gillespie D.A. (2017). PERK inhibits DNA replication during the Unfolded Protein Response via Claspin and Chk1. Oncogene.

[42] Alfieri C., Zhang S., Barford D. (2017). Visualizing the complex functions and mechanisms of the anaphase promoting complex/cyclosome (APC/C). Open Biol..

[43] Cappell S.D., Mark K.G., Garbett D., Pack L.R., Rape M., Meyer T. (2018). EMI1 switches from being a substrate to an inhibitor of APC/CCDH1 to start the cell cycle. Nature.

[44] Chen M., Gutierrez G.J., Ronai Z.A. (2012). The anaphase-promoting complex or cyclosome supports cell survival in response to endoplasmic reticulum stress. PLoS ONE.

[45] Zeman M.K., Cimprich K.A. (2014). Causes and consequences of replication stress. Nat. Cell Biol..

[46] Malhotra J.D., Miao H., Zhang K., Wolfson A., Pennathur S., Pipe S.W., Kaufman R.J. (2008). Antioxidants reduce endoplasmic reticulum stress and improve protein secretion. Proc. Natl. Acad. Sci. USA.

[47] Sun S.Y. (2010). N-acetylcysteine, reactive oxygen species and beyond. Cancer Biol. Ther..

[48] Wang F.M., Galson D.L., Roodman G.D., Ouyang H. (2011). Resveratrol triggers the pro-apoptotic endoplasmic reticulum stress response and represses pro-survival xbp1 signaling in human multiple myeloma cells. Exp. Hematol..

[49] Bellezza I., Giambanco I., Minelli A., Donato R. (2018). Nrf2-Keap1 signaling in oxidative and reductive stress. Biochim. Biophys. Acta-Mol. Cell Res..

[50] Forman H.J., Davies K.J.A., Ursini F. (2014). How do nutritional antioxidants really work: Nucleophilic tone and para-hormesis versus free radical scavenging in vivo. Free Radic. Biol. Med..

[51] Farkhondeh T., Folgado S.L., Pourbagher-Shahri A.M., Ashrafizadeh M., Samarghandian S. (2020). The therapeutic effect of resveratrol: Focusing on the Nrf2 signaling pathway. Biomed. Pharmacother..

[52] da Silva D.A., Correia T.M.L., Pereira R., da Silva R.A.A., Augusto O., Queiroz R.F. (2020). Tempol reduces inflammation and oxidative damage in cigarette smoke-exposed mice by decreasing neutrophil infiltration and activating the Nrf2 pathway. Chem. Biol. Interact..

[53] Jannatifar R., Parivar K., Roodbari N.H., Nasr-Esfahani M.H. (2020). The effect of N-acetyl-cysteine on NRF 2 antioxidant gene expression in asthenoteratozoospermia men: A clinical trial study. Int. J. Fertil. Steril..

[54] Maio N., Lafont B.A.P., Sil D., Li Y., Bollinger J.M.J., Krebs C., Pierson T.C., Linehan W.M., Rouault T.A. (2021). Fe-S cofactors in the SARS-CoV-2 RNA-dependent RNA polymerase are potential antiviral targets. Science.

[55] Brown V.A., Patel K.R., Viskaduraki M., Crowell J.A., Perloff M., Booth T.D., Vasilinin G., Sen A., Schinas A.M., Piccirilli G. (2010). Repeat dose study of the cancer chemopreventive agent resveratrol in healthy volunteers: Safety, pharmaco-kinetics, and effect on the insulin-like growth factor axis. Cancer Res..

[56] Patel K.R., Brown V.A., Jones D.J., Britton R.G., Hemingway D., Miller A.S., West K.P., Booth T.D., Perloff M., Crowell J.A. (2010). Clinical pharmacology of resveratrol and its metabolites in colorectal cancer patients. Cancer Res..

[57] Zemelko V.I., Grinchuk T.M., Domnina A.P., Artzibasheva I.V., Zenin V.V., Kirsanov A.A., Bichevaia N.K., Korsak V.S., Nikolsky N.N. (2011). Multipotent mesenchymal stem cells of desquamated endometrium: Isolation, characterization and use as feeder layer for maintenance of human embryonic stem cell lines. Tsitologiya.

[58] Vang O., Ahmad N., Baile C.A., Baur J.A., Brown K., Csiszar A., Das D.K., Delmas D., Gottfried C., Lin H.Y. (2011). What is new for an old molecule? systematic review and recommendations on the use of resveratrol. PLoS ONE.

