# Supplementary material for: Induction of Premature Cell Senescence Stimulated by High Doses of Antioxidants Is Mediated by Endoplasmic Reticulum Stress
Source: Int J Mol Sci. 2021 Oct 31;22(21):11851. doi: 10.3390/ijms222111851 (PMC8584632; doi:10.3390/ijms222111851)
Supplement: Supplementary file 1 [file ijms-22-11851-s001.zip › Supplement_Figures_rev1.pdf]

Supplement

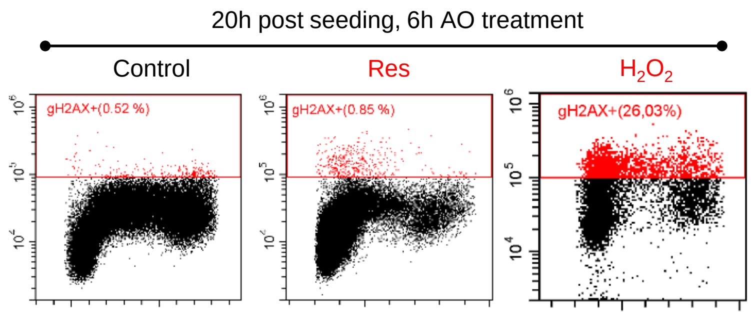

a)

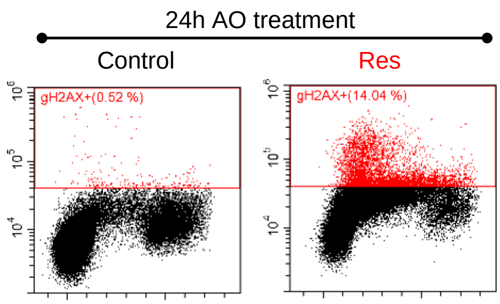

b)

Figure S1. MSCs treated with resveratrol in the early S phase of the cycle accumulate DNA breaks: representative dot plots of the cell cycle distributions of MSCs stained with γH2AX antibodies after 6-hour (a) and 24-hour (b) incubations with resveratrol. Cells treated with H_2O_2 for one hour (a) were used as a positive control. Abbreviations: AO, antioxidants; Res, resveratrol; MSCs, human mesenchymal stem cells, line#2804

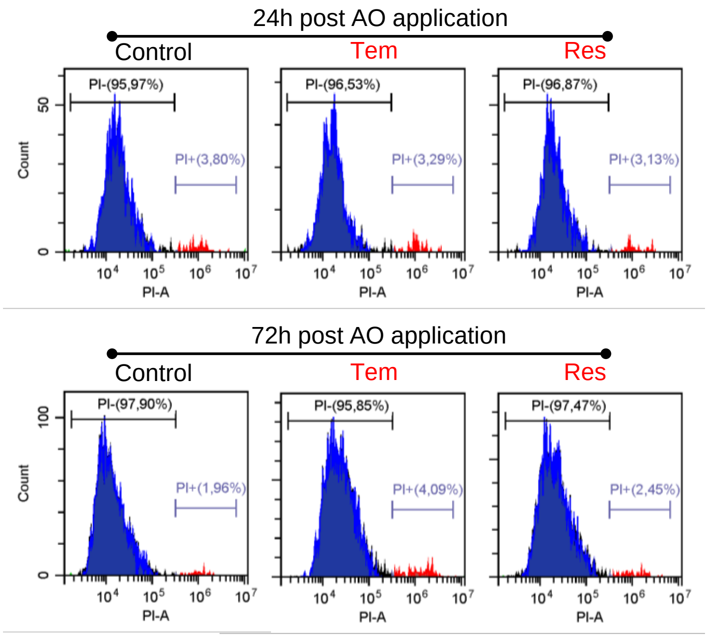

a)

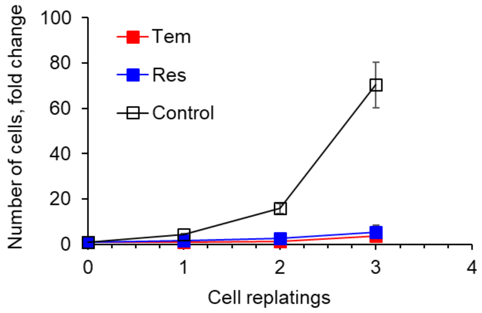

b)

Figure S2. MSCs treated with AOs in the early S phase of the cycle preserve viability but lose proliferative capacity: (a) representative histograms of PI-stained control MSCs and MSCs treated with AOs for 24 hours, the analysis was performed prior to washing off AOs and two days later; (b) growth curves for the control MSCs and MSCs treated with AOs for 24 hours (cells were replated and counted every 4 days). Abbreviations: AO, antioxidants; Res, resveratrol; Tem, Tempol; PI, propidium iodide; MSCs, human mesenchymal stem cells, line#2804

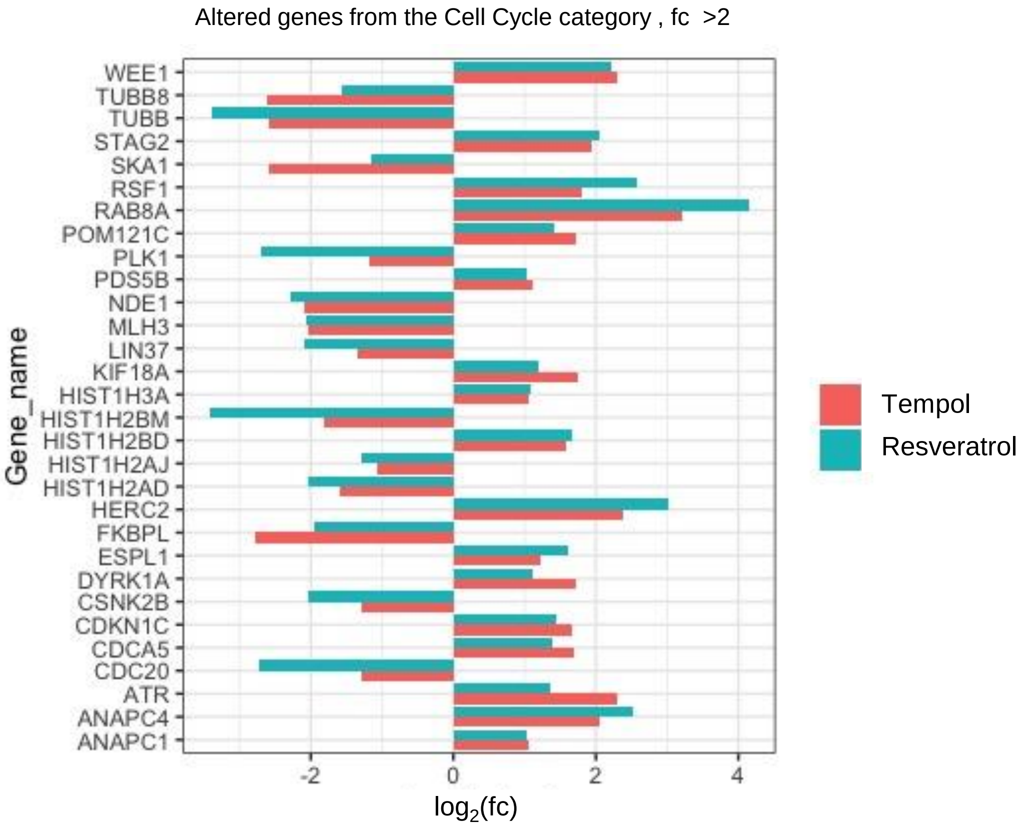

Figure S3. AO treatments result in the S phase progression slowdown: downregulation of DNA replication and mitosis regulators (WEE1, ANAPC4, ANAPC1, PDS5B, KIF18A, HERC2, DYRK1A, CDKN1C, CDCA5, ATR) in AO-treated MSCs.

Abbreviations: fc, fold change of gene expression in Tempol- and resveratrol-treated MSCs

Altered genes from the *Gene Expression* category, fc >2

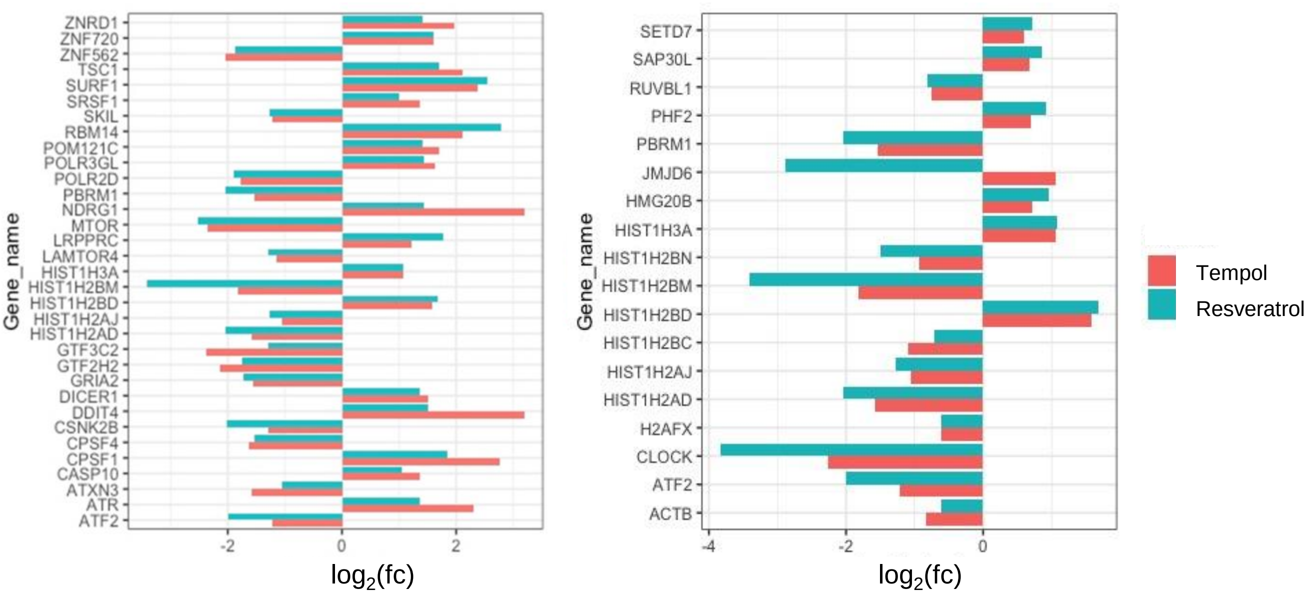

Figure S4. AO treatments result in the downregulation of transcription activators (ATF2, CLOCK, PBRM1, RUVBL1, POLR2D, GTF3C2, GTF2H2) and upregulation of transcription repressors (SAP30L, DICER1). Abbreviations: fc, fold change of gene expression in Tempol- and resveratrol-treated MSCs

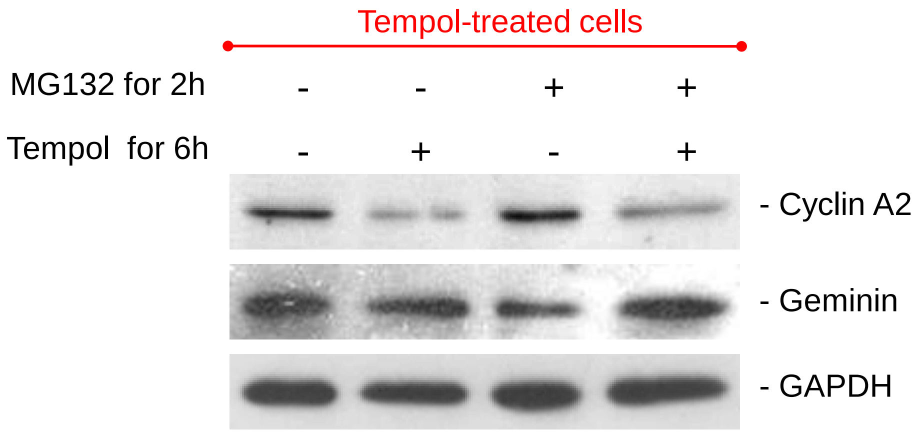

Figure S5. Tempol treatments cause instability of the key regulatory proteins of the S phase. Level of cyclin A2 and geminin proteins in the control and Tempol--treated MSCs incubated with Tempol for 6 hours with or without proteosome inhibitor MG132. MG132 was applied at 4 hours post Tempol addition and partly stabilized the level of cyclin A2 and geminin proteins in Tempol-treated cells, in contrast to the control cells.

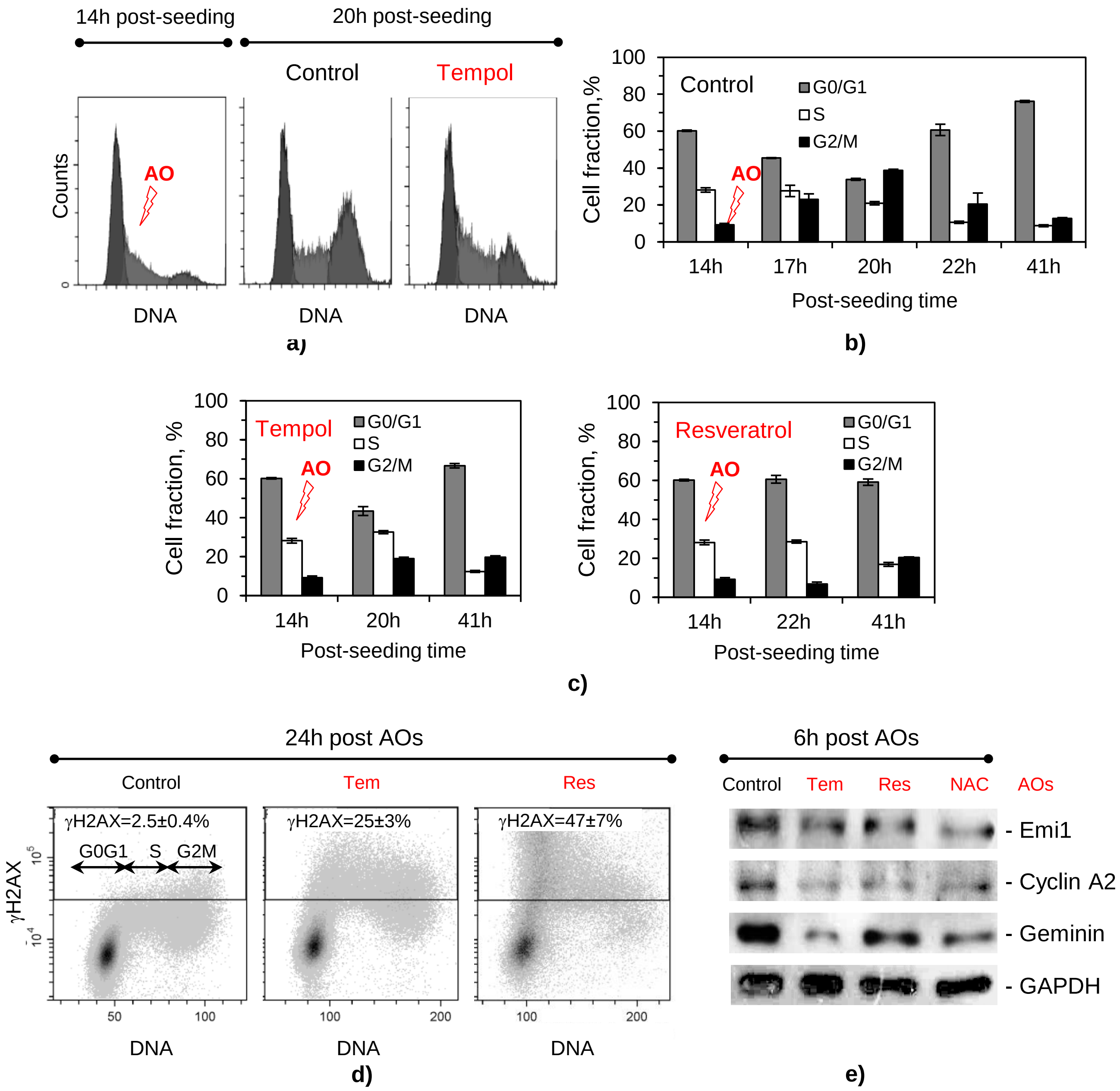

Figure S6. Fibroblasts treated with AOs in the S phase of the cell cycle respond to AO treatments in the similar to MSC cultures way. (a) Flow cytometry histograms of the control and Tempol-treated fibroblasts reveal slowdown of S-phase progression induced by AO treatment. (b, c) Cell cycle distributions measured by flow cytometry at different time points post cell seeding in the control (b) and AO-treated (c) cells. The data show that AO-treated fibroblasts progress through the S phase slowly and eventually are accumulated in the G_2/M phase. Data are presented as the mean ± SD of three independent experiments. (d) Flow cytometry studies of the γH2AX+ cell distribution among the cell cycle phases in the control and AO-treated fibroblasts exposed to AOs for 24 hours. Dotplots show accumulation of DNA strand breaks in cells replicating their DNA. (e) Immunoblot analysis of cyclin A2, geminin and Emi1 proteins in the AO-treated fibroblasts lysed at 6 h post AO treatment reveals the low level of the S phase regulatory proteins. Abbreviations: AOs, antioxidants, namely Tempol (2 mM), resveratrol (40 μM), N-acetyl-L-cysteine (NAC, 20mM).

Table S2. Primers used for qPCR analysis.

Symbol	Primer sequence	PCR product size (bp)	NCBI Reference Sequence
GRP78	F 5’ TAGCGTATGGTGCTGCTGTC 3’<br>R 5’ TGACACCTCCCACAGTTTCA 3’	117	NM_005347.5
HERPUD1	F 5’ TCCAAAGCAGGAAAAACGGC 3’<br>R 5’ CCTCAGGATACTGTCCCCGA 3’	137	NM_014685.4
EDEM1	F 5’ ACAACTACATGGCTCACGCC 3’<br>R 5’ AGATTTGAAGGGTCCCCGC 3’	91	NM_014674.3
DNAJC3	F 5’ CCACACACCTTTCCTCCTCT 3’<br>R 5’ GCAGATCCACCAGGACTAGC 3’	113	NM_006260.5
DNAJB9	F 5’ AGTCGGAGGGTGCAGGATATT 3’<br>R 5’ TTGATTTGGCGCTCTGATGC 3’	153	NM_012328.3
HSP47	F 5’ ATGCAGAAGAAGGCTGTTGC 3’<br>R 5’ GGCCTTGTTCTTGTCAATGG 3’	117	NM_001207014.3
ACTIN	F 5’ GCACTCTTCCAGCCTTCC 3’<br>F 5’ AGAAAGGGTGTAACGCAACTAAG 3’	385	NM_001101.5
