# Supplementary material for: Induction of Premature Cell Senescence Stimulated by High Doses of Antioxidants Is Mediated by Endoplasmic Reticulum Stress
Source: Int J Mol Sci. 2021 Oct 31;22(21):11851. doi: 10.3390/ijms222111851 (PMC8584632; doi:10.3390/ijms222111851)
Supplement: Supplementary file 1 [file ijms-22-11851-s001.zip › Supplement_Table S1_Lyublinskaya.pdf]

Table S1. Set of significant (p-value ≤ 0.1, Bonferroni correction) differentially expressed genes common for Tempol and resveratrol groups (referred to as AO-DEG list).

N	Gene symbol	Gene full name	Fold change (Tempol)	Fold change (Resveratrol)	Pathway (REACTOME)	Pathway Category (REACTOME)
1	PDK2	Pyruvate Dehydrogenase Kinase 2	0.51	0.51	The citric acid (TCA) cycle and respiratory electron transport	Metabolism
2	NADK	NAD Kinase	0.75	0.7	Nicotinate metabolism	Metabolism
3	GOPC	Golgi Associated PDZ And Coiled-Coil Motif Containing	1.3	1.93	RHO GTPases regulate CFTR trafficking	Signal Transduction
4	ANAPC4	Anaphase Promoting Complex Subunit 4	4.17	5.76	Mitotic Metaphase and Anaphase	Cell Cycle
					Cell Cycle Checkpoints	Cell Cycle
					Synthesis of DNA	DNA Replication
					Senescence-Associated Secretory Phenotype (SASP)	Cellular responses to external stimuli
5	MTFR1	Mitochondrial Fission Regulator 1	5.05	4.71	Release of apoptotic factors from the mitochondria	Programmed Cell Death
6	MLTK	Mitogen-Activated Protein Kinase Kinase Kinase 20	2.54	2.67	ERK/MAPK targets	Signal Transduction
					Deactivation of the beta-catenin transactivating complex	Signal Transduction
					KSRP (KHSRP) binds and destabilizes mRNA	Metabolism of RNA
7	GADD45B	Growth Arrest And DNA Damage Inducible Beta	6.58	11.34	TP53 Regulates Transcription of Cell Cycle Genes	Gene expression (Transcription)
					FOXO-mediated transcription of cell cycle genes	Gene expression (Transcription)
8	CABIN1	Calcineurin Binding Protein 1	0.11	0.09	DNA Damage/Telomere Stress Induced Senescence	Cellular responses to external stimuli
9	SRRD	SRR1 Domain Containing	3.08	3.94	NA	NA
10	XBP1	X-Box Binding Protein 1	2.32	2.13	XBP1(S) activates chaperone genes	Metabolism of proteins
					Unfolded Protein Response (UPR)	Metabolism of proteins
11	CDK5RAP1	CDK5 Regulatory Subunit Associated Protein 1	0.68	0.75	NA	NA
12	MAGT1	Magnesium Transporter 1	32.03	40.88	Asparagine N-linked glycosylation	Metabolism of proteins
					Miscellaneous transport and binding events	Transport of small molecules

13	SLC39A14	Solute Carrier Family 39 Member 14	1.75	1.36	Transport of bile salts and organic acids, metal ions and amine compounds	Transport of small molecules
14	CCNE1	Cyclin E1	1.5	2.24	Mitotic G1 phase and G1/S transition	Cell Cycle
					Synthesis of DNA	DNA Replication
15	COPE	COPI Coat Complex Subunit Epsilon	2.21	2.96	COPI-mediated anterograde transport	Vesicle-mediated transport
					Intra-Golgi and retrograde Golgi-to-ER traffic	Vesicle-mediated transport
16	SEC61B	SEC61 Translocon Subunit Beta	0.83	0.8	Insertion of tail-anchored proteins into the endoplasmic reticulum membrane	Protein localization
					SRP-dependent cotranslational protein targeting to membrane	Metabolism of proteins
17	DUSP3	Dual Specificity Phosphatase 3	1.19	0.9	MAPK targets/ Nuclear events mediated by MAP kinases	NA
18	NDUFC1	NADH:Ubiquinone Oxidoreductase Subunit C1	0.61	0.62	Respiratory electron transport, ATP synthesis by chemiosmotic coupling and heat production by uncoupling proteins.	Metabolism
19	CHD4	Chromodomain Helicase DNA Binding Protein 4	0.71	0.71	PTEN Regulation	Signal Transduction
					NGF-stimulated transcription	Signal Transduction
					Chromatin organization	Chromatin organization
					RNA Polymerase I Transcription	Gene expression (Transcription)
					ERCC6 (CSB) and EHMT2 (G9a) positively regulate rRNA expression	Gene expression (Transcription)
					Regulation of TP53 Activity through Acetylation	Gene expression (Transcription)
20	TIMMDC1	slocase Of Inner Mitochondrial Membrane Domain Containi	2.53	3.09	Respiratory electron transport, ATP synthesis by chemiosmotic coupling and heat production by uncoupling proteins.	Metabolism

21	RNF7	Ring Finger Protein 7	0.67	0.76	Neddylation	Metabolism of proteins
22	CDC20	Cell Division Cycle 20	0.41	0.15	RHO GTPases Activate Formins	Signal Transduction
					Mitotic Prometaphase	Cell Cycle
					Mitotic Metaphase and Anaphase	Cell Cycle
23	MLH3	MutL Homolog 3	0.24	0.24	Meiotic recombination	Cell Cycle
24	UBIAD1	UbiA Prenyltransferase Domain Containing 1	0.72	0.8	Metabolism of vitamin K	Metabolism
25	PYROXD1	Pyridine Nucleotide-Disulphide Oxidoreductase Domain 1	4	3.75	NA	NA
26	VAPB	VAMP Associated Protein B And C	2.6	1.79	Sphingolipid metabolism	Metabolism
27	NUP85	Nucleoporin 85	0.68	0.54	Mitotic Prometaphase	Cell Cycle
					Mitotic Metaphase and Anaphase	Cell Cycle
					RHO GTPases Activate Formins	Signal Transduction
					Glucose metabolism	Metabolism
					Gene Silencing by RNA	Gene expression (Transcription)
28	UBR4	Ubiquitin Protein Ligase E3 Component N-Recognin 4	1.52	1.33	Class I MHC mediated antigen processing & presentation	Immune System
29	DNAJB9	DnaJ Heat Shock Protein Family (Hsp40) Member B9	3.54	2.09	XBP1(S) activates chaperone genes	Metabolism of proteins
30	MPP1	Membrane Palmitoylated Protein 1	0.66	0.74	Regulation of PLK1 Activity at G2/M Transition	Cell Cycle
					Transcriptional Regulation by E2F6	Gene expression (Transcription)
					COPI-independent Golgi-to-ER retrograde traffic	Vesicle-mediated transport
					Sphingolipid de novo biosynthesis	Metabolism
31	ABCB7	ATP Binding Cassette Subfamily B Member 7	3.38	6.9	ABC-family proteins mediated transport	Transport of small molecules
					Cytosolic iron-sulfur cluster assembly	Metabolism
32	MPRIP	Myosin Phosphatase Rho Interacting Protein	1.37	1.34	NA	NA
33	POSTN	Periostin	1.6	1.77	NA	NA
34	STARD13	StAR Related Lipid Transfer Domain Containing 13	0.14	0.12	Signaling by Rho GTPases	Signal Transduction
35	TESPA1	Thymocyte Expressed, Positive Selection Associated 1	0.48	0.51	NA	NA

36	HNRNPA1	Heterogeneous Nuclear Ribonucleoprotein A1	0.63	0.64	Processing of Capped Intron-Containing Pre-mRNA	Metabolism of RNA
					Signaling by FGFR2	Signal Transduction
37	KLHL36	Kelch Like Family Member 36	0.19	0.17	NA	NA
38	PDCL	Phosducin Like	1.62	2.22	Protein folding	Metabolism of proteins
39	APTX	Aprataxin	0.7	0.72	UMOylation of DNA damage response and repair proteins	Metabolism of proteins
					SUMOylation of transcription factors	Metabolism of proteins
					Regulation of TP53 Expression	Gene expression (Transcription)
					Transcriptional Regulation by VENTX	Gene expression (Transcription)
					Downregulation of SMAD2/3:SMAD4 transcriptional activity	Signal Transduction
40	DUSP5	Dual Specificity Phosphatase 5	2.66	2.41	RAF-independent MAPK1/3 activation	Signal Transduction
41	TIMM23	Translocase Of Inner Mitochondrial Membrane 23	0.7	0.69	Mitochondrial protein import	Protein localization
42	IMPA2	Inositol Monophosphatase 2	1.21	1.53	Inositol phosphate metabolism	Metabolism
43	SRD5A1	Steroid 5 Alpha-Reductase 1	1.97	1.97	Metabolism of steroid hormones	Metabolism
44	RSU1	Ras Suppressor Protein 1	1.48	1.63	Cell junction organization	Cell-Cell communication
45	ADD3	Adducin 3	0.4	0.33	Miscellaneous transport and binding events	Transport of small molecules
46	PKNOX1	PBX/Knotted 1 Homeobox 1	2.83	0.44	NA	NA
47	MFSD8	Major Facilitator Superfamily Domain Containing 8	3.46	4.09	NA	NA
48	RPS14	Ribosomal Protein S14	0.76	0.78	Eukaryotic Translation Initiation	Metabolism of proteins
					Eukaryotic Translation Elongation	Metabolism of proteins
					Eukaryotic Translation Termination	Metabolism of proteins
					SRP-dependent cotranslational protein targeting to membrane	Metabolism of proteins
					Major pathway of rRNA processing in the nucleolus and cytosol	Metabolism of RNA
					Nonsense-Mediated Decay (NMD)	Metabolism of RNA

					Selenoamino acid metabolism	Metabolism
					Response of EIF2AK4 (GCN2) to amino acid deficiency	Cellular responses to external stimuli
					Signaling by ROBO receptors	Developmental Biology
49	NGFRAP1	Brain Expressed X-Linked 3	0.68	0.76	p75 NTR receptor-mediated signalling	Signal Transduction
50	TMEM170A	Transmembrane Protein 170A	2.15	2.1	NA	NA
51	PLK1	Polo Like Kinase 1	0.44	0.15	Mitotic Prometaphase	Cell Cycle
					Mitotic Metaphase and Anaphase	Cell Cycle
					Mitotic G2-G2/M phases	Cell Cycle
					RHO GTPases Activate Formins	Signal Transduction
					Cilium Assembly	Organelle biogenesis and maintenance
52	CTNNB1	Catenin Beta 1	1.91	3.34	Transcriptional Regulation by VENTX	Gene expression (Transcription)
					Transcriptional regulation by RUNX3 (RNA Polymerase II Transcription)	Gene expression (Transcription)
					TCF dependent signaling in response to WNT	Signal Transduction
					Degradation of beta-catenin by the destruction complex	Signal Transduction
					Beta-catenin independent WNT signaling	Signal Transduction
					RHO GTPases activate IQGAPs	Signal Transduction
					Signaling by VEGF	Signal Transduction
					Apoptotic execution phase	Programmed Cell Death
					Incretin synthesis, secretion, and inactivation	Metabolism of proteins
					Cell junction organization	Cell-Cell communication
53	SMIM4	Small Integral Membrane Protein 4	2.7	7.08	NA	NA
54	HIST1H1E	H1.4 Linker Histone, Cluster Member	0.44	0.31	DNA Damage/Telomere Stress Induced Senescence	Cellular responses to external stimuli

					Apoptotic execution phase	Programmed Cell Death
55	SDC2	Syndecan 2	1.74	1.64	Regulation of Insulin-like Growth Factor (IGF) transport and uptake by Insulin-like Growth Factor Binding Proteins (IGFBP) (ER)	Metabolism of proteins
					EPH-Ephrin signaling	Developmental Biology
					Glycosaminoglycan metabolism	Metabolism
56	RPLP2	Ribosomal Protein Lateral Stalk Subunit P2	0.74	0.78	Eukaryotic Translation Initiation	Metabolism of proteins
					Eukaryotic Translation Elongation	Metabolism of proteins
					Eukaryotic Translation Termination	Metabolism of proteins
					SRP-dependent cotranslational protein targeting to membrane	Metabolism of proteins
					Major pathway of rRNA processing in the nucleolus and cytosol	Metabolism of RNA
					Nonsense-Mediated Decay (NMD)	Metabolism of RNA
					Selenoamino acid metabolism	Metabolism
					Response of EIF2AK4 (GCN2) to amino acid deficiency	Cellular responses to external stimuli
					Signaling by ROBO receptors	Developmental Biology
57	C5orf30	Macrophage Immunometabolism Regulator	15.23	14.09	TNFR1-induced NFkappaB signaling pathway	Signal Transduction
					Regulation of TNFR1 signaling	Signal Transduction
58	RTKN2	Rhotekin 2	0.25	0.27	RHO GTPases Activate Rhotekin and Rhophilins	Signal Transduction
59	TM2D3	TM2 Domain Containing 3	0.88	1.47	NA	NA
60	SP140L	SP140 Nuclear Body Protein Like	0.34	0.39	NA	NA
61	MCRS1	Microspherule Protein 1	0.87	0.67	Nucleotide Excision Repair	DNA Repair
					Deubiquitination	Metabolism of proteins
					Chromatin organization	Chromatin organization

62	LAMTOR4	e Endosomal/Lysosomal Adaptor, MAPK And MTOR Activat	0.45	0.41	mTOR signalling	Signal Transduction
					Energy dependent regulation of mTOR by LKB1-AMPK	Signal Transduction
					PTEN Regulation (PIP3 activates AKT signaling)	Signal Transduction
					Amino acids regulate mTORC1	Cellular responses to external stimuli
					Macroautophagy	Autophagy
63	TMEM63A	Transmembrane Protein 63A	0.23	0.23	Neutrophil degranulation	Immune System
64	HIST1H2AD	H2A Clustered Histone 7	0.33	0.24	SASP Pol1 Gene silencing SIRT1 rRNA DNAmetilation PRC2 Telomere DNA damage	NA
					Amyloid fiber formation	Metabolism of proteins
65	ARHGAP11A	Rho GTPase Activating Protein 11A	2.84	1.98	Signaling by Rho GTPases	Signal Transduction
66	TMEM199	Transmembrane Protein 199	0.18	0.17	trans-Golgi Network Vesicle Budding	NA
67	RNU6-219P	RNA, U6 Small Nuclear 219, Pseudogene	0.25	0.19	NA	NA
68	PPAPDC1A	Phospholipid Phosphatase 4	0.68	0.58	NA	NA
69	MIR635	MicroRNA 635	0.2	0.35	NA	NA
70	LDHAP5	Lactate Dehydrogenase A Pseudogene 5	1.69	0.68	NA	NA
71	LDHAP4	Lactate Dehydrogenase A Pseudogene 4	1.63	0.65	NA	NA
72	RPL21P1	Ribosomal Protein L21 Pseudogene 1	25.49	33.13	NA	NA
73	RPL24P7	RPL24 Pseudogene 7	0.61	0.71	NA	NA
74	RP11-475C16.1	RP11-475C16.1	0.76	0.73	NA	NA
75	RP11-69L16.5	RP11-69L16.5	0.72	0.73	NA	NA
76	RNU6-1203P	RNA, U6 Small Nuclear 1203, Pseudogene	5.07	4.18	NA	NA
77	AC010546.1	AC010546.1	4.23	8.67	NA	NA
78	RP1-228H13.1	RP1-228H13.1	2.44	2.54	NA	NA
79	RP11-307O1.1	RP11-307O1.1	0.65	0.74	NA	NA
80	RP11-145H9.3	RP11-145H9.3	3.36	2.13	NA	NA
81	RP11-206L10.11	RP11-206L10.11	2.55	6.33	NA	NA
82	RP11-553N16.1	RP11-553N16.1	0.86	0.89	NA	NA
83	HMGN2P3	h Mobility Group Nucleosomal Binding Domain 2 Pseudoger	0.67	0.7	NA	NA
84	RPL24P8	RPL24 Pseudogene 8	0.71	0.71	NA	NA
85	RP11-490G8.1	RP11-490G8.1	0.71	0.71	NA	NA
86	TMEM141	Transmembrane Protein 141	0.21	1.26	NA	NA
87	RP11-73G16.3	RP11-73G16.3	0.32	0.31	NA	NA

88	RP1-68D18.2	RP1-68D18.2	0.67	0.68	NA	NA
89	SNORD112	Small Nucleolar RNA, C/D Box 112	0.13	0.21	NA	NA
90	snoU13	Small Nucleolar RNA, C/D Box 13 Pseudogene 1	2.77	2.58	NA	NA
91	Y_RNA	RNA, Ro60-Associated Y3	0.16	0.14	NA	NA
92	RP11-121G22.3	RP11-121G22.3	0.22	0.22	NA	NA
93	RP11-467L19.11	RP11-467L19.11	0.65	0.43	NA	NA
94	PTP4A2P1	PTP4A2 Pseudogene 1	1.4	1.46	NA	NA
95	RP11-264B14.2	RP11-264B14.2	4.02	8.74	NA	NA
96	RP11-254F7.3	RP11-254F7.3	0.17	0.16	NA	NA
